# Non-genetic factors and breast cancer: an umbrella review of meta-analyses

**DOI:** 10.1186/s12885-024-12641-8

**Published:** 2024-07-26

**Authors:** Anneza Yiallourou, Katerina Pantavou, Georgios Markozannes, Antonis Pilavas, Andrea Georgiou, Andria Hadjikou, Mary Economou, Neophytos Christodoulou, Konstantinos Letsos, Elina Khattab, Chrystalleni Kossyva, Maria Constantinou, Melanie Theodoridou, Daniele Piovani, Konstantinos Κ. Tsilidis, Stefanos Bonovas, Georgios K. Nikolopoulos

**Affiliations:** 1 Medical School, University of Cyprus, P.O. Box 20537, Nicosia, 1678 Cyprus; 2 Department of Epidemiology and Biostatistics, School of Public Health, Imperial College London, London, SW7 2AZ UK; 3 Department of Hygiene and Epidemiology, University of Ioannina School of Medicine, Ioannina, 45110 Greece; 4 Royal Papworth Hospital, Papworth Rd, Trumpington, Cambridge, CB2 0AY UK; 5 Department of Biomedical Sciences, Humanitas University, Milan, 20072 Italy; 6 IRCCS Humanitas Research Hospital, Milan, 20089 Italy

**Keywords:** Breast cancer, Non-genetic factors, Overview of reviews, Umbrella review, Systematic review, Meta-analysis

## Abstract

**Background:**

Previous research has found associations between various non-genetic factors and breast cancer (BrCa) risk. This study summarises and appraises the credibility of the available evidence on the association between non-genetic factors and BrCa risk.

**Methods:**

We conducted an umbrella review of meta-analyses. Medline, Scopus, and the Cochrane databases were systematically searched for meta-analyses examining non-genetic factors and BrCa incidence or mortality. The strength of the evidence was graded in four categories (i.e., *weak, suggestive, highly suggestive, convincing*).

**Results:**

A total of 781 meta-analyses from 280 publications were evaluated and graded. We included exposures related to anthropometric measurements, biomarkers, breast characteristics and diseases, diet and supplements, environment, exogenous hormones, lifestyle and social factors, medical history, medication, reproductive history, and pregnancy. The largest number of examined associations was found for the category of diet and supplements and for exposures such as aspirin use and active smoking. The statistically significant (*P-*value < 0.05) meta-analyses were 382 (49%), of which 204 (53.4%) reported factors associated with increased BrCa risk. Most of the statistically significant evidence (*n* = 224, 58.6%) was graded as *weak*. *Convincing* harmful associations with heightened BrCa risk were found for increased body mass index (BMI), BMI and weight gain in postmenopausal women, oral contraceptive use in premenopausal women, increased androstenedione, estradiol, estrone, and testosterone concentrations, high Breast Imaging Reporting and Data System (BIRADS) classification, and increased breast density. *Convincing* protective factors associated with lower BrCa risk included high fiber intake and high sex hormone binding globulin (SHBG) levels while *highly suggestive* protective factors included high 25 hydroxy vitamin D [25(OH)D] levels, adherence to healthy lifestyle, and moderate-vigorous physical activity.

**Conclusions:**

Our findings suggest some highly modifiable factors that protect from BrCa. Interestingly, while diet was the most studied exposure category, the related associations failed to reach higher levels of evidence, indicating the methodological limitations in the field. To improve the validity of these associations, future research should utilise more robust study designs and better exposure assessment techniques. Overall, our study provides knowledge that supports the development of evidence-based BrCa prevention recommendations and guidance, both at an individual level and for public health initiatives.

**Trial registration:**

PROSPERO CRD42022370675.

**Supplementary Information:**

The online version contains supplementary material available at 10.1186/s12885-024-12641-8.

## Background

Breast cancer (BrCa) is the most commonly diagnosed cancer worldwide, with an estimated 2.3 million cases and 685,000 deaths in 2020 [1]. Incidence and death rates of female BrCa remain high in developed countries [1] and rapidly increase in transitioning ones (countries with lower Human Development Index). The latter could be attributed to the fact that countries with growing economies have been experiencing significant changes of lifestyle and sociocultural patterns, which, along with the increasing involvement of women in the industrial workforce, have resulted in changes of the prevalence of BrCa risk factors [1, 2].

Approximately 10% of all female BrCa cases are familial and linked to specific highly penetrant gene mutations (e.g., *BRCA1*, *BRCA2*) [3]. However, the highest proportion of cases are attributed to both low penetrant genetic and non-genetic factors [3]. For example, menopausal status is an important non-genetic factor that determines BrCa risk [4]. Variations in premenopausal and postmenopausal BrCa incidence and mortality across different countries are associated with income differences as well as with the differential distribution of distinct molecular features and risk factors in each of the two menopausal statuses [4]. In addition, BrCa is classified into molecular subtypes based on whether BrCa cells grow in response to female hormones (i.e., estrogen, progesterone) or growth factors [5]. Stratification of women based on non-genetic risk factors for BrCa is of paramount importance for developing more effective risk reduction strategies as well as for targeted risk- stratified BrCa screening programmes [6].

There is a large number of systematic reviews and meta-analyses on non-genetic factors (including obesity, hormone levels, alcohol consumption, and smoking) and their association with BrCa risk and mortality [7–10]. However, the results are often contradictory and subject to biases. A few umbrella reviews (i.e., reviews of systematic reviews and meta-analyses), which examined certain types of non-genetic exposures, also included BrCa as one of the studied outcomes [11–16]. However, to our knowledge, there has been no systematic effort to summarise and evaluate the robustness of evidence on non-genetic risk factors for BrCa.

Therefore, in view of the large, and often contradictory, amount of published evidence on non-genetic risk factors for BrCa incidence and mortality, we aimed to summarise and evaluate the findings of systematic reviews and meta-analyses in this field, following an umbrella review methodology. The added value of the present umbrella review is that it offers a comprehensive and deep understanding of the aetiology of BrCa by integrating findings from various systematic reviews and meta-analyses, thereby providing a thorough and reliable assessment of the evidence regarding non-genetic factors and risk of BrCa.

## Methods

A standardised methodology based on a predefined internal protocol was registered in PROSPERO (CRD42022370675). The findings are reported according to the PRIOR [17] (Preferred Reporting Items for Overviews of Reviews) recommendations (Additional file 1 – PRIOR Checklist).

### Search strategy

We identified relevant systematic reviews and meta-analyses investigating the association of any non-genetic factor and BrCa incidence and/or mortality. We searched Medline (via PubMed), Scopus, and the Cochrane database for systematic reviews from inception to October 31st, 2022. The following search algorithm was used: ((Breast OR mammary) AND (cancer* OR neoplasm* OR malignant* OR tumour* OR tumor* OR carcinoma* OR adenocarcinoma*)) AND (meta-analysis OR "systematic review" OR systematic review). The full strategy can be found in the supplement (Additional file 1 – Search strategy).

### Eligibility criteria

We included systematic reviews and meta-analyses published in English that studied the association of any non-genetic exposure with female BrCa incidence or mortality due to BrCa as the primary cause of death (when mortality was reported as proxy for incidence in primary studies) among healthy individuals at risk for BrCa. Studies involving women with pre-existing breast cancer investigating survival outcomes following cancer diagnosis were excluded. There were no restrictions depending on publication status such as preprints. However, certain types of publication (e.g., books, commentary, letters) were not evaluated as they were considered unlikely to provide sufficient data for inclusion in our analysis. We only included papers that had performed a systematic literature search; meta-analysis papers without a systematic search of the literature were excluded. We considered meta-analyses if they included at least two independent primary studies. Sub-analyses in a meta-analysis that included only one study were excluded. Finally, we excluded any (otherwise eligible) publications when they did not provide effect estimates and their corresponding confidence intervals (CIs) or some other measure, such as standard errors or *P*﻿-values, for the individual studies in the meta-analyses, or enough data to reproduce them. Systematic reviews focusing on the association between genes or genetic markers and BrCa risk or on the survival of BrCa cases were not considered. The exclusion criteria are presented in the supplement (Additional file 1 – Exclusion criteria).

Title, abstract, and full text screening was performed in duplicate by 9 authors (AP, AG, AH, ME, KL, EK, CK, MC, MT). Conflicts were resolved by discussion with other team members (AY, KP, GM, GKN) until consensus was reached. In case there were multiple overlapping meta-analyses, we chose only one for our umbrella review, based on the following algorithm: First, we selected the most recent systematic review and meta-analysis. If another meta-analysis had been conducted within 5 years from the date of publication of the most recent one, we chose the one with the largest number of individual studies and largest number of participants, and the most comprehensive one (i.e., the one evaluating the largest number of different comparisons for the risk factor in question). Quality was assessed using the AMSTAR tool [18], which also served as an additional selection criterion if the preceding criteria were comparable.

### Data extraction

Data extraction was performed by 7 authors (AP, CK, EK, KL, KP, MC, MT) using a predefined extraction form in Excel. The validity of data extraction was evaluated by another 4 independent authors (AG, AH, GM, ME). The extracted information from each eligible publication included the first author’s last name, year of publication, BrCa types with respect to hormone receptors and human epidermal growth factor receptor 2 (HER2), BrCa stage, examined risk factors, number of studies and estimates included in meta-analyses, characteristics of the study populations (e.g., origin, menopausal status, other characteristics), meta-analysis metric (odds ratio, risk ratio, hazard ratio, etc.; if the meta-analysis metric was not clear from the original publication we used the summary metric as reported in the meta-analysis), meta-analysis method (fixed- or random-effects), summary effect estimates and 95% CIs, and the level of control for potential confounders performed in the studies included in the meta-analysis (adjusted, not adjusted). Our umbrella review described in detail and graded only meta-analyses that were based on studies with adjusted estimates. Meta-analyses of primary studies with crude summary effect estimates (either in totality, dubbed as unadjusted, or partially, dubbed as mixed) are included in the Additional files 1 and 2 to allow for a comprehensive review of the non-genetic risks examined in the literature. However, they were not graded to avoid grading associations at high risk of bias. Within each of the studied associations we extracted data on the first author of the primary study included in the meta-analysis along with the year of publication, study design, and effect estimate with corresponding 95% CI (or any other measure of variation of the effect estimate reported), number of cases and population size (in cohort studies) or the numbers of cases and controls (in case–control studies).

### Statistical analysis

This study adopted the methodological approach used in umbrella reviews [19, 20]. Briefly, for each association included in this umbrella review we calculated the summary effect estimate and the corresponding 95% CI using the inverse variance weighted random-effects model [21] due to the expected clinical and methodological heterogeneity across primary studies included in the meta-analytical associations and for consistency in the application of the evidence grading criteria. We assessed the proportion of total variability in effect estimates due to between-study heterogeneity of each meta-analysis using the I^2^ metric of inconsistency [22] and we also calculated the 95% prediction intervals, which show the range in which the effect estimate of a new study in the future is expected to lie [23]. The possibility of small study effects was assessed using the Egger’s regression asymmetry test [24] (with a significance threshold of 0.10), and based on whether the summary estimate was larger in magnitude than the effect estimate of the largest (i.e., most precise; smallest standard error) primary study included in that meta-analysis. Finally, we used the excess significance test to evaluate whether the observed number of studies in the meta-analysis that presented a nominally significant result (*P*-value < 0.05) was different from the expected number of studies with significant results [22]. The expected number of statistically significant studies was estimated based on the sum of the statistical power of each individual study, which is a function of the number cases and the total sample size. For meta-analyses in which this information was missing for at least 20% of the primary studies, the excess significance test was not performed.

### Quality assessment

The quality of the eligible systematic reviews/meta-analyses was evaluated using the AMSTAR tool [18]. AMSTAR critically appraises the quality of systematic reviews and meta-analyses using 11 items and focusing on key methodological issues. Due to the large number of meta-analyses and primary studies included in this umbrella review, the risk of bias was not assessed individually for each primary study considered in each meta-analysis.

### Grading of the evidence

The certainty of the evidence (i.e., the confidence in the effect estimate) was graded in a four-point scale (i.e., *weak, suggestive, highly suggestive,* and *convincing* evidence) using certain statistical criteria [19, 20] (Table 1) in accordance with previous umbrella reviews [12, 25, 26]. Associations that did not present at least a statistically significant result (*P*-value < 0.05) in the random-effects model were “non-significant” and, thus, they were not graded.

**Table 1:** Statistical criteria used for grading the evidence in the umbrella review of meta-analyses on non-genetic risk factors and breast cancer

Criteria	Robustness of evidence category			
	***Convincing***	***Highly suggestive***	***Suggestive***	***Weak***
Number of cases	1000	1000	1000	-
Random effects *P*-value	< 10^–6^	< 10^–6^	< 10^–3^	< 0.05
Largest study^a^	Statistically significant	Statistically significant	-	-
I^2^	< 50%	-	-	-
95% Confidence interval	Null value is excluded^b^	-	-	-
Small study effects^c^	Absent	-	-	-
Excess significance bias	Absent	-	-	-

^a^Study with the smallest standard error in the meta-analysis

^b^Null value: 0 for continuous and 1 for binary outcomes

^c^Small study bias was based on the *P*-value of the Egger’s regression asymmetry test (*P*-value < 0.1) and the random effects summary estimate was larger compared to the point estimate of the largest study in a meta-analysis

## Results

### Literature search

**Fig. 1:**
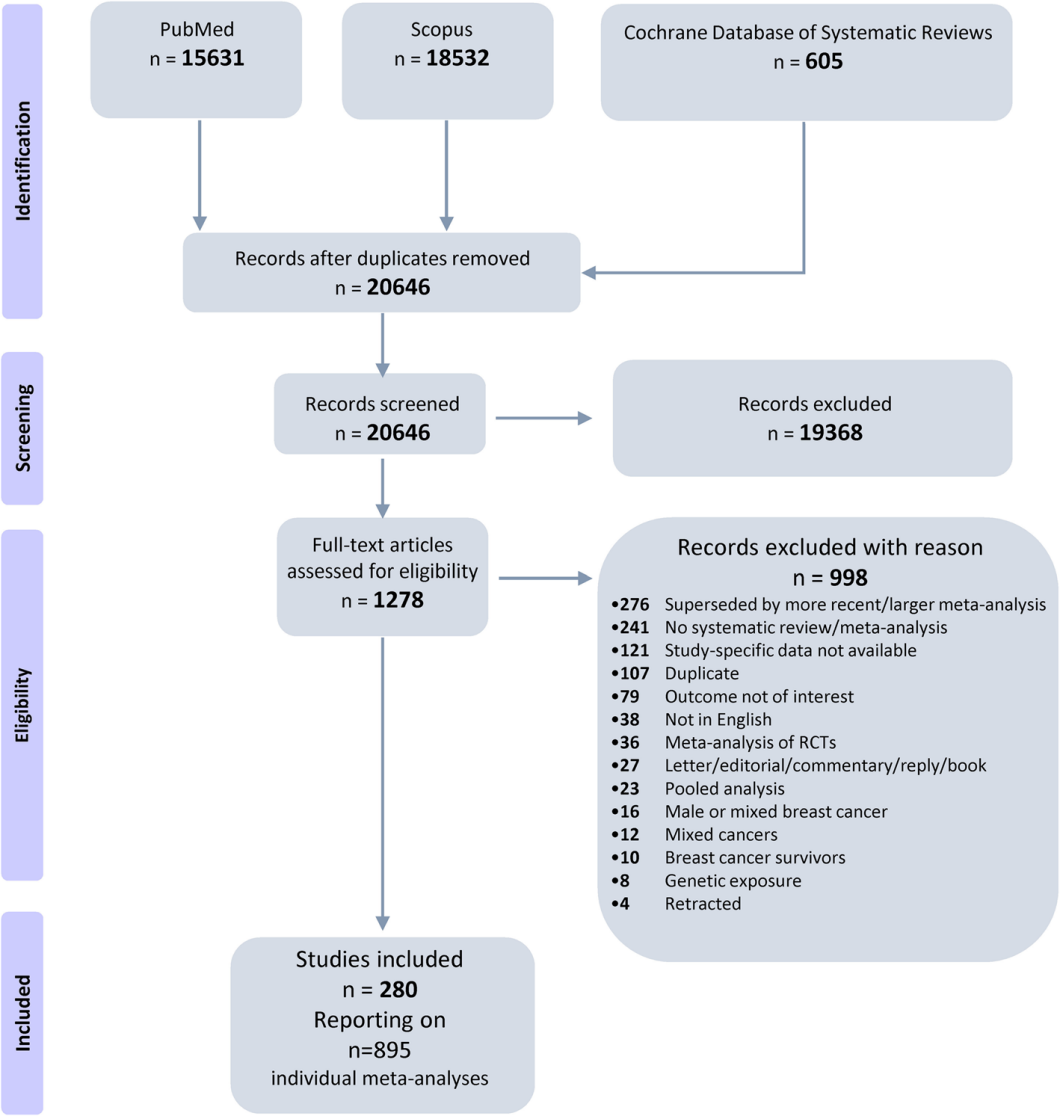
Flowchart of the literature search and study selection process in the umbrella review of meta-analyses on non-genetic risk factors and breast cancer

The search algorithm yielded a total of 20,646 unique citations across the three databases (Fig. 1), of which 1,278 were deemed potentially eligible. After excluding 998 publications in the full-text screening phase (Additional file 2—Table S1), 280 publications [7–10, 27–302] were included in our review, presenting a total of 895 meta-analytic associations of non-genetic factors with BrCa (Additional file 2—Tables S2 and S3). Of these, 781 were meta-analyses of studies with adjusted estimates while 114 meta-analyses included (either in totality or partially) primary studies with crude summary effect estimates. The publication dates ranged between 1995 and 2022.

### Quality assessment

Methodological quality, as assessed using AMSTAR, varied across the 280 publications considered in our umbrella review (Additional file 2—Table S4). The median score was 8 (interquartile range: 6 to 9). Common flaws were the absence of reference to a published protocol (*n* = 178, 63.6%), the use of publication status as an inclusion criterion (*n* = 213, 76.1%), and the use of methodological quality in formulating conclusions and recommendations (*n* = 165, 5.89%). In about 25% (*n* = 69) of the publications there was no reference to a comprehensive literature search.

### Description of the results

In the following sections, only the 781 meta-analyses with adjusted estimates are considered. A brief description of the 114 meta-analyses including (either in totality or partially) primary studies with crude summary effect estimates is presented in the Additional file 1 and the Additional file 2—Table S3.

The median number of included studies in the meta-analyses was 7 (range 2 to 80). Six-hundred-and-thirty-nine (81.8%) meta-analytic estimates pertained to overall BrCa incidence or mortality (with 7 estimates being specific to BrCa ­related mortality), while 131 (16.8%) focused on BrCa molecular subtypes, i.e., estrogen (ER), progesterone (PR), HER2, luminal A and B, and triple-negative, and 11 (1.4%) specifically to the locoregional spread, i.e., in-situ, invasive, localised, non-localised. Most associations (*n* = 568, 72.7%) pertained to the general population, while 176 (22.5%) associations pertained to menopausal status and 37 (4.8%) to specific populations (i.e., country-, race-, mutation-, parity-, or hormone replacement therapy-specific).

### Overview of the available evidence

**Fig. 2:**
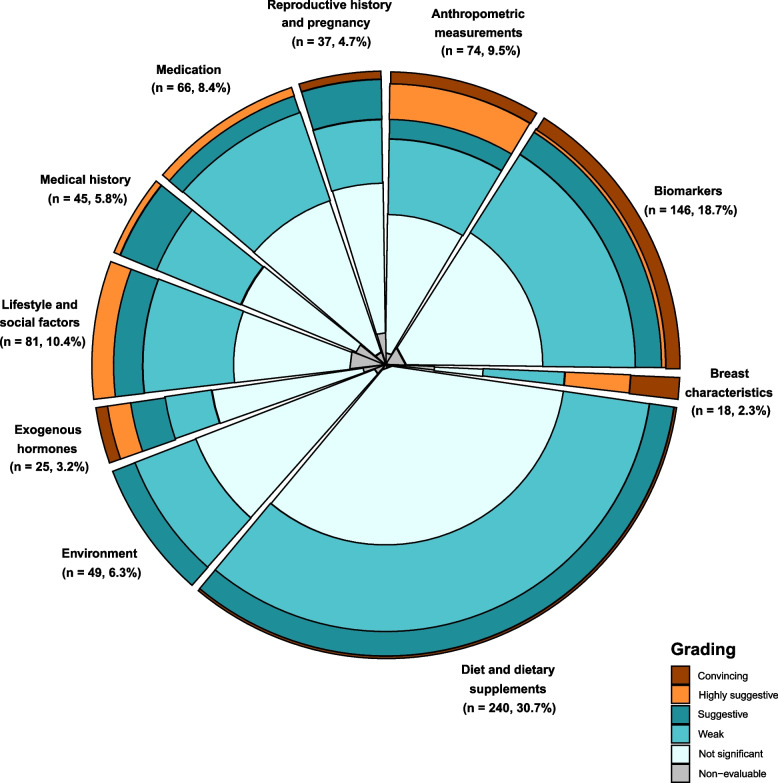
Summary of the classification of non-genetic factors examined in the 781 meta-analyses and the distribution of the grading for their association with breast cancer risk

The identified non-genetic factors were classified in 11 categories (anthropometric measurements, biomarkers, breast characteristics, diet and dietary supplements, environment, exogenous hormones, lifestyle and social factors, medical history, medication, reproductive history, and pregnancy; Fig. 2). All meta-analyses in the family history–consanguinity category were based on unadjusted estimates; thus, this category was not further considered in the evidence assessment.

Most of the 781 meta-analyses with adjusted estimates (Additional file 2—Table S2) that examined the association of non-genetic factors with BrCa risk were classified in the diet and supplements category (*n* = 240, 30.7%; Fig. 2). Biomarkers were examined by 18.7% (*n* = 146) of the meta-analyses with adjusted estimates, and lifestyle and social factors by 10.4% (*n* = 81) of them. A large number of meta-analyses were found for aspirin use (*n* = 22, 2.8%), body mass index (BMI) in adulthood or childhood (*n* = 17, 2.2%), night shift work (*n* = 16, 2.1%), weight gain (*n* = 16, 2.1%), Mediterranean dietary pattern (*n* = 13, 1.7%), body weight (*n* = 12, 1.5%), and breastfeeding¸ bisphosphonates use, and oral contraceptives (OC) use (each *n* = 11, 1.4%).

About half (*n* = 382; 49%) of the 781 meta-analyses were statistically significant (random-effects *P*-value < 0.05). Of these, 178 (46.6%) associations indicated a decreased risk of BrCa, and 204 (53.4%) an increased risk of BrCa. At a *P-*value threshold of 10^–3^, 166 (21.3%) meta-analyses were significant (of these, 103 [62%] indicated an increased risk), whereas for a *P-value* threshold of 10^–6^, 81 (10.4%) meta-analyses remained significant (*n* = 59, 72.8% indicated an increased risk).

High heterogeneity (I^2^ ≥ 50%) was found in 346 (44.3%) meta-analyses, and in 181 (52.3%) among those with statistically significant results (*P*-value < 0.05). The 95% prediction intervals excluded the null value (i.e., 1 for binary outcomes) in 69 (8.9%) associations. Evidence of small study effects was observed in 121 (15.5%) meta-analyses. Evidence of excess significance bias was observed in 83 (10.6%) meta-analyses. However, for almost half of the meta-analyses (*n* = 370; 47.4%), excess significance bias was not estimated, as in these meta-analyses, information was missing for at least 20% of the primary studies.

### Evidence for non-genetic factors and BrCa risk

A comprehensive description of the evidence for the association between the non-genetic factors and BrCa risk from meta-analyses with adjusted estimates is shown in the Additional file 1.

### Strength of epidemiological evidence

Figure 2 illustrates the classification of non-genetic factors examined in the 781 meta-analyses and the distribution of the grading for their association with BrCa. Tables 2 and 3 summarise the confidence in the effect estimates for protective and harmful non-genetic factors for BrCa (Table S2) and for BrCa receptor-related outcomes [estrogen receptor positive/negative (ER ±), progesterone receptor positive/negative (PR ±), human epidermal growth factor receptor 2 (HER2), luminal, triple negative], reaching at least *weak* evidence (Table S3).

**Table 2:** Strength of epidemiologic evidence for protective and harmful non-genetic factors for breast cancer (risk, mortality, invasive, non-invasive, in situ, and localized) in the umbrella review of relevant meta-analyses

	**Decreases risk**				**Increases risk**			
	***Convincing***	***Highly suggestive***	***Suggestive***	***Weak***	***Weak***	***Suggestive***	***Highly suggestive***	***Convincing***
**Anthropometric measurements**		BMI (at ages 18–30 years)^a^ [7]		BMI^b^ [7], BMI (at ages 18–30 years)^b^ [7], Weight loss^b^ [7], Weight loss^a^ [7]	Birth length [93], Birth weight [144, 159, 190, 210, 282], Fat mass (kg)^a^ [94], WC^b^ [7], WC^a,b^ [7], Weight gain (MHT ever)^a^ [7], WHR^b^ [7]	Fat mass (%)^a^ [94], Weight gain (MHT never)^a^ [7], Weight gain since menopause^a^ [179]	BMI^a^ [7], Height [300], Weight gain^a^ [7, 179]	BMI gain^a^ [7], Weight gain (HRT non-users)^a^ [286]
**Biomarkers**	SHBG^a^ [182]	25(OH)D (per 5 nmol/L) [123]	EPA [87], n-3/n-6 PUFA ratio (serum) [87], 25(OH)D (high vs low) [123], SHBG [182]	A-carotene [239], Adiponectin [10], B-carotene (per 50 μg/dL) [239], Calcium [47], Carotenoids [239], Daidzein [73], Genistein [73], HDL-C (high vs low) [211], DHA [87], DPA [87], n-3 LC-PUFA [87], Lutein [239], Lutein/ zeaxanthin [239], Lycopene [239], MBP/MiBP [69], n-3 LC-PUFA (erythrocytes) [87], n-3/n-6 PUFA ratio (serum, diet) [269], PLP [260], TNFa (high vs low) [10], Total Antioxidant Capacity [136]	Androstenedione^b^ [182]_,_ C-peptide (fasting/non-fasting) [275], C-peptide/insulin [115], CRP [295], DEHP [69], d-ROM (pre-diagnostic) [96], Estrone, urinary [182], Estrone sulfate [182], Fasting glucose (non-diabetic) [244], Iron [109], MUFA^a^ [27], Palmitic acid^a^ [27], Resistin [10], 25(OH)D deficiency (< 10 ng/mL) [102], Testosterone, free^b^ [182]	Androstenedione^a^ [182]_,_ Anti-thyroid antibodies [236], DHEAS [182], DHEAS^b^ [182], Estradiol, free [182], Estradiol, urinary [182], Estrone [182], IGF-1^a^ [214], Testosterone^b^ [182]	Estradiol [182]	Androstenedione [182], Estradiol^a^ [182], Estrone^a^ [182], Testosterone [182], Testosterone^a^ [182], Testosterone, free [182]
**Breast characteristics**					Breast density (high vs low)^a,b^ [59], Wolfe grade (P2 + Dy vs N1 + P1) [49]		Wolfe grade (P1, P2 and Dy vs N1) [49]	BIRADS classification (D vs B) [215]
**Diet and dietary supplements**	Total fiber^a^ [134]		B-carotene (dietary) [231], Calcium (dietary) [41], Folate (dietary) [274], Fruit fiber (high vs low) [134], Isoflavones [58], Mushrooms (per 1 g/day) [273], Soy (high vs low) [143], Soy food/isoflavones^a,b^ [271], Tofu [125], Total fiber [55], Vitamin A [230], Vitamin C (dietary/ supplemental; high vs low) [153] *Dietary patterns*: Mediterranean [70], Prudent [98]	A-carotene (dietary) [231], Aclylamide^a^ [132], B-carotene [239], Calcium (dietary/ supplemental) [41], Carrots [91], Citrus fruits [253], Cheese (per 30 g/day) [157], Cruciferous vegetables [241], Dairy or milk [35], Flavan-3-ols [58], Flavones [200], Flavonols [200], Folate (per 100 mg/day) [156], Fruits [157, 238], Fruits/ vegetables [157, 238], Insoluble fiber (high vs low) [134], Light colored vegetables^c^ [283], Marine n-3 PUFA [257], Methionine [260], Miso [82], Mushrooms^a^ [273], Olive oil [191], Onion [208], Selenium [36], Skimmed milk (per 200 g/day) [57], Soluble fiber (high vs low) [134], Soy (per 30 g/day) [157], Soy food/isoflavones [120], Total allium vegetables [208], Total dairy food [228], Total fiber^b^ [134], Total tea [267], Vegetables [157], Vegetable fiber (high vs low) [134], Vitamin B2 [133], Vitamin C (dietary) [153], Vitamin C (dietary/ supplemental), Whole grain (per 50 g/day) [92] *Dietary patterns*: Mediterranean^a^ [70], Prudent^b^ [98]	Alcohol (Former vs non-drinkers) [183], Cholesterol [43], Dietary inflammatory index^b^ [187], Glycemic Index [72, 193], Heme iron [109], Processed meat [157, 178], Red meat [157, 183], Sugar-sweetened beverages^b^ [161], Total fat [270], Total meat [157] *Dietary patterns*: Drinker [192], Early-life energy restriction [50], Western [98]	Dietary inflammatory index [187], Red meat^b^ [220], Wine [40]		Alcohol (≤ 1 drink/day vs non-drinking) [64]
**Environment**			Solar radiation (≥ 1 vs < 1 h/day in summer months) [124]	Mono-benzyl phthalate [168], Mono-2-isobutyl phthalate [168]	EMF (extremely low frequency) [32] Hair chemicals (rinse) [158], Hair dye (any and permanent) [158], NO2 (long-term) [173], PCB-187 and PCB-99 exposure [34], PCB-exposure (group II) [30], Urban residential environment [291]	EMF (occupational/ residential) [259], Environmental tobacco smoke (non-smokers) [46], PCB-183 exposure [34]		
**Exogenous hormones**				IVF [258]	Estrogen [183], Levonorgestrel-releasing intrauterine system [128], OC^b^ [71]	MHT^a^ [243], OC^b^ [71]	Estrogen-progestin therapy [85], OC (use before FFTP vs non-use)^a^ [71]	OC (< 50 years, parous)^b^ [71]
**Lifestyle and social factors**	-	Healthy lifestyle (WCRF/AICR score) [130], Moderate-vigorous recreational PA^a,b^ [54]	Lifetime PA [113], Occupational PA^a^ [7], PA (at ages 5–30 years) [113]	Leisure time PA [222], Lifetime PA^b^ [113], PA (at ages 5–30 years)^a,b^ [113]	Artificial light^a,b^ [180], Artificial light (indoor) [180], Circadian disrupting [280], Low sitting time^a^ [7], Marital status (unmarried vs married, lifelong single vs married) [131], Night shift work (yes vs no, < 10 years vs never, ≥ 15 years vs never, more than 5 times a week vs never) [164, 177, 189, 204, 256], Sedentary behavior (per 1 h/day and occupational) [142, 296], Sleep-disordered breathing [164], Start of night shift work (before menopause vs never) [204], Life striking events [105]	Active smoking (per 1 year and per pack-year) [8], Sedentary behavior (yes vs no and high vs low occupational) [172, 296]	Active smoking (ever vs never) [8], Educational level [116]	
**Medical history**			Bariatric surgery [167], Risk-reducing salpingo-oophorectomy (BRCA1 and 2 mutations careers) [198]	Anorexia nervosa (nulliparous and at age > 20 years) [110], BMD (lumbar spine) [99], Huntington's disease [266], Hyporthyroidism^a,b^ [217], Migraine [196]	Diabetes mellitus (Type 2) [242], Hyperthyroidism^a^ [217], Metabolic syndrome (invasive BrCa and NCEP-ATP III) [121], Periodontal disease [97], Schizophrenia [266], Tonsillectomy [209]	Hypertension [111], Hyperthyroidism [217]	Diabetes mellitus [242]	
**Medication**			Aspirin (use vs non-use) [166] Bisphosphonates (use vs non-use) [155]	DPP-4 inhibitors (Type 2 diabetes mellitus women) [81], Aspirin (use vs non-use^a^, regular dose vs non-use or duration ≥ 3 years vs non-use) [166], Ibuprofen [126], Bisphosphonates (specific years of use vs non-use [114], Etidronate [155]	Calcium channel blockers [169], Digoxin (≥ 3 years use vs non-use) [65], Diuretics [169], Antipsychotic [212]	Antibiotic [145] Cardiac glycosides [56]	Digoxin (use vs non-use) [65]	
**Reproductive history and pregnancy**			Breastfeeding [248]	Breastfeeding^c^ [62]	Parity [183], Paternal age at delivery [93]			

*Abbreviations*: *25(OH)D* 25 hydroxy vitamin D, *BIRADS* Breast imaging reporting and database system, *BMD* Bone mineral density, *BMI* Body mass index, *BrCa* Breast cancer, *BRCA1 or 2* Breast cancer type 1 or 2 susceptibility gene, *CRP* C-reactive protein, *DEHP* Di(2- ethylhexyl) phthalate, *DHA* Docosahexaenoic acid, *DPP-4* Dipeptidyl peptidase 4, *DPA* Docosapentaenoic acid, *d-ROM* Derivatives of reactive oxygen metabolites, *EMF* Electromagnetic fields, *EPA* Eicosapentaenoic acid, *FFTP* First full-term pregnancy, *HDL-C* High-density lipoprotein cholesterol, *HRT* Hormone replacement therapy, *IGF-1* Insulin-like growth factor 1, *IVF* In vitro fertilization, *MBP* Mono-n-butyl phthalate, *MHT* Post-menopausal hormone therapy, *MiBP* Mono-iso-butyl phthalate, *MUFA* Monounsaturated fatty acids, *n-3 LC-PUFA* n-3 long chain polyunsaturated fatty acids, *NCEP-ATP III* National Cholesterol Education Program—Adult Treatment Panel III, *OC* Oral contraceptives, *PA* Physical activity, *PCB* Polychlorinated biphenyl, *PCB-exposure (group II)* PCB-exposure (potentially antiestrogenic and immunotoxic, dioxin-like), *PLP* Pyridoxal 5’-phosphate, *PUFA* Polyunsaturated fatty acids, *SHBG* Sex hormone binding globulin, *TNFa* Tumor necrosis factor-a, *WC* Waist circumference, *WCRF/AICR score* World Cancer Research Fund/American Institute for Cancer Research, *WHR* Waist-to-hip ratio

^a^Postmenopausal

^b^Premenopausal

^c^Specific population (e.g., Asian, Japanese, Korean, Indian, Iranian, Japanese, Western parous/ nulliparous, NAT2 slow genotype carriers)

**Table 3:** Strength of epidemiologic evidence for protective and harmful non-genetic factors for breast cancer receptor-related outcomes (ER ± , PR ± , HER2, luminal, triple negative) in the umbrella review of relevant meta-analyses

	**Decreases risk**				**Increases risk**			
	***Convincing***	***Highly suggestive***	***Suggestive***	***Weak***	***Weak***	***Suggestive***	***Highly suggestive***	***Convincing***
**Anthropometric measurements**				*ER* + */PR* + *:* Body weight^b^ [137]	*ER* + */PR* + *:* Body weight [137], *ER-/PR* + : Body weight^a^ [137] *ER-/PR-:* Weight gain [226], Weight gain (high vs low) [226]	*ER* + */PR* + *:* Weight gain (high vs low [226] and per 5 Kg^b^ [7])	*ER* + *:* BMI^a^ [7] *ER* + */PR* + *:* BMI^a^ [7], Body weight^a^ [137]	*PR* + *:* BMI^a^ [7]
**Biomarkers**				*ER* + *:* SHBG [182]	*ER-/PR-*: Estradiol [182] *ER-*: Estradiol, free [182] *ER* + *PR-*: Testosterone [182] *ER* + *PR* + : Testosterone [182] *ER-:* Testosterone, free [182]			
**Breast characteristics**					*ER-:* Breast density (≥ 75% vs < 25% and 50%-74% vs < 25%)^c^ [247]		*ER* + *:* Breast density (≥ 75% vs < 25%)^c^ [247]	*ER-:* Breast density (50%-74% vs < 25% and 25%-49% vs < 25%)^c^ [247]
**Diet and dietary supplements**			*ER- PR-:* Prudent pattern [98]	*ER-/PR* + *:* Mediterranean pattern [70] *ER* + *PR* + *:* Prudent pattern [98]	*ER* + */PR-:* Alcohol [9] *ER* + *PR* + *:* Mediterranean pattern [70]	*ER* + */PR* + *:* Alcohol [9]		
**Lifestyle and social factors**				*ER-:* Vigorous PA^a^ [7]	*ER* + *:* Artificial light [180] *PR* + *:* Night shift work [204]			
**Medication**				*ER* + *:* Aspirin [166] *PR* + *:* Aspirin [166] *ER* + */PR* + *:* Aspirin [166]			*ER* + *:* Digoxin [65]	
**Reproductive history and pregnancy**	*ER* + */PR* + *:* Age at menarche [60]		*ER* + */PR* + *:* Age at menarche^b^ [60] *ER-/PR-:* Breastfeeding [28] *Luminal A:* Parity [184] *Luminal B:* Parity [184]	*ER* + */PR* + *:* Age at menarche^a^ [60], Breastfeeding [28] *ER-/PR-:* Age at menarche^b^ [60] *Triple-negative:* Breastfeeding [45]	*ER* + */PR* + *:* Age at first pregnancy [45]			

*Abbreviations*: *BMI* Body mass index, *ER* Estrogen receptor, *OC* Oral contraceptives, *PA* Physical activity, *PR* Progesterone receptor

^a^premenopausal

^b^postmenopausal

^c^Western population

Seventeen associations (4.4% of 382 meta-analyses with significant results; 2.2% of 781 meta-analyses with adjusted estimates) were graded as *convincing.* These included three protective associations [older age at menarche (BrCa, ER + /PR + BrCa) [60], higher sex hormone binding globulin (SHBG) [182], and higher total fiber [134]] and 15 associations supporting an increased risk of BrCa [alcohol consumption [64], higher BMI (PR + BrCa) [7], BMI gain [7] and weight gain in postmenopausal women [286], Breast Imaging Reporting and Data System (BIRADS) classification for breast density (D versus B) [215], breast density (25%-49% and 50%-74% vs < 25%; ER- BrCa) [247], higher levels of sex hormones including androstenedione, estradiol, estrone, and testosterone in the general population and in postmenopausal women [182], and oral contraceptive (OC) use in premenopausal women [71].

*Highly suggestive* epidemiological evidence was found for 26 associations (6.8% of 382 meta-analyses with significant results; 3.3% of 781 meta-analyses with adjusted estimates). Of these, an increased risk of BrCa was found for higher BMI in postmenopausal women (BrCa, ER + and ER + /PR + BrCa) [7], body weight in postmenopausal women (ER + /PR + BrCa) [137], height [300], weight gain in postmenopausal women [7, 179], estradiol levels [182], Wolfe grade (P1, P2, Dy versus N1) [49], breast density (≥ 75% vs < 25%; ER + BrCa) [247], estrogen-progestin therapy [85] and digoxin use (BrCa, ER + BrCa) [65], ever active smoking [8], higher educational level [116], and diabetes mellitus [242]. In contrast, higher early adult BMI in postmenopausal women [7], 25 hydroxy vitamin D [25(OH)D] levels [123], adherence to the World Cancer Research Fund/American Institute for Cancer Research Recommendations (WCRF/AICR) score [130], and moderate-vigorous recreational physical activity (PA) [54] had a protective role.

Sixty-eight associations (17.8% of 382 meta-analyses with significant results; 8.7% of 781 meta-analyses with adjusted estimates) were graded as *suggestive*, while 224 (58.6% of the 382 significant; 28.7% of the 781 total) statistically significant meta-analyses were graded as *weak*. Finally, 47 (12.3% of the 382 significant; 6% of the 781 total) nominally significant meta-analyses that did not provide the necessary data for grading (number of cases and excess significance bias) were not considered (Additional file 2—Table S2).

## Discussion

### Principal findings

This large umbrella review of meta-analyses systematically summarised and critically appraised the epidemiological evidence for the association between non-genetic risk factors and female BrCa. Overall, 895 associations (781 meta-analyses of studies with adjusted estimates) were considered, reporting exposures related to anthropometric measurements, biomarkers, breast characteristics and diseases, diet and supplements, environmental parameters, exogenous hormones, factors associated with pregnancy or birth, lifestyle and social factors, medical history, medication, and reproductive history. The highest number of examined associations was found for the category of diet and supplements and for exposures such as aspirin use and active smoking.

Most of the examined associations were either non-significant (51%) or were supported by *weak* evidence (28.7%). Only about 5.5% of the associations (11.3% of those with statistically significant results) were graded as *convincing* or *highly suggestive*. These meta-analyses supported that alcohol consumption, high BMI (BrCa and ER + , PR + , ER + /PR + BrCa), high body weight (BrCa, ER + /PR + BrCa) and body weight gain in postmenopausal women, high height, P1/ P2/ DY Wolfe grade and high BIRADS/ Breast density classification (BrCa and ER-, ER + BrCa), OC use in premenopausal women, ever active smoking, high androstenedione, estradiol, estrone, and testosterone levels, estrogen-progestin therapy use, high educational level, diabetes mellitus, and digoxin use (BrCa, ER + BrCa) were associated with increased BrCa risk. On the other hand, high BMI at ages 18–30 years in premenopausal women, adherence to the WCRF/AICR score, high moderate-vigorous recreational physical activity in postmenopausal women, menarche at an older age (BrCa, ER + /PR + BrCa), increased total fiber intake, increased blood levels of 25(OH)D, and high levels of SHBG were found to prevent from BrCa. Of note, the associations of body weight and breast density with BrCa, despite reaching high levels of evidence, had a low score in the AMSTAR quality assessment.

### Strengths and weaknesses in relation to other studies

In the current era of abundant scientific research, umbrella reviews have emerged as a crucial tool to consolidate and synthesize evidence across entire research domains. It is expected that a few associations covered in our extensive analysis might have already been partially addressed in existing umbrella reviews [12, 14, 303–307]. Nonetheless, our review stands out as the most comprehensive to date, offering a thorough mapping and assessment of all non-genetic risk factors for BrCa. Of note, the association between human papillomavirus infection and BrCa that was graded as convincing in a recent umbrella [305] review was not assessed in ours because it was based on unadjusted estimates. We considered this type of meta-analysis to have a high likelihood of bias.

Our study findings align significantly with existing evidence, reinforcing associations previously acknowledged as robust or reaching high evidence levels, such as alcohol [14], BMI [12], physical activity [11], dietary uptake of fiber [308], diabetes [13], sex hormones, and age of menarche [309]. Additionally, our research highlights new associations, including those for digoxin [65], 25(OH)D, breast density, and healthy lifestyle measured as a WCRF/AICR score. However, we did observe a limited number of associations for which our evidence level conflicted with that from previous studies. For example, coffee consumption, one of the most studied exposures in other umbrella reviews [14, 310, 311], reached “probable” levels of evidence in one of them [310]. However, there was no statistically significant association in our umbrella review similarly to the rest of the reviews in this topic [14, 311]. That review, which found “probable” levels of evidence for coffee consumption, followed a grading approach that allowed for higher levels of evidence to be reached although the included meta-analytical association was not statistically significant [310].

### Biological plausibility

The biological mechanisms of the association between BrCa risk and height, obesity, physical activity, diabetes, and sex hormones are related. Height is related to the onset of puberty, which is affected by endogenous estrogens, whose role on BrCa has been very well documented [312–314]. On the other hand, there might be a causal association between height and BrCa, in which various genetic and non-genetic factors affect height and, subsequently, BrCa risk through a shared biological pathway [300, 315]. As an example, insulin-like growth factor 1 (IGF-I) has been proven to play a pivotal role in cell proliferation enhancement and apoptosis suppression, while it is also considered to be a major determinant of growth and height [214, 316]. In postmenopausal women, synthesis of estrogens takes place in the adipose tissue, whereas in premenopausal women the major source of estrogens are the ovaries. Obesity in postmenopausal women leads to increased conversion of androgens to estrogens, and, as result, to the promotion of cell proliferation and the inhibition of apoptosis. Furthermore, obesity has been associated with insulin resistance and hyperinsulinemia, which downregulates sex hormone binding globulin production, and, thus, results in increased levels of circulating estradiol. On the other hand, it has been reported that more frequent anovulatory cycles among obese premenopausal women [317, 318], and faster clearance rate of free estrogen in the liver among obese compared to lean women [319] may lead to lower levels of both estrogen and progesterone [320]. The protective effect of high moderate-vigorous physical activity against BrCa in postmenopausal women is most probably explained by the fact that exercise helps to prevent obesity.

The association of BrCa with diabetes could be explained through similar pathways such as the activation of the insulin pathway, the activation of insulin- like growth factor pathway, as well as the regulation of sex hormones. Moreover, hyperglycemia has been associated with increased levels of IGF-I and inflammatory cytokines, resulting in direct and indirect effects on cancer cells proliferation, apoptosis, and metastasis. Insulin promotes the expression of insulin receptors in BrCa cells and thus leads to the malignant transformation of breast epithelial cells. Increased insulin resistance on the other hand could cause higher levels of insulin and, as a result, increased androgen synthesis and decreased estrogen production. High SHBG levels have been shown to have a protective role against BrCa. Apart from the apparent function of the regulation of free sex hormones levels, SHBG seems to act as a direct mediator for cell-surface signaling, cellular delivery, and the biologic action of sex hormones, which results in the regulation of the bioavailable fraction of circulating estradiol [321, 322]. Through these unique features, SHBG reduces BrCa cell growth and proliferation [323, 324].

Alcohol is classified as a Group 1 human carcinogen by the International Agency for Research on Cancer [325] and is acknowledged by the World Health Organization as one of the major modifiable risk factors for breast cancer [326]. Alcohol consumption may contribute to BrCa development through various pathways, including hormonal modulation, DNA damage, oxidative stress, immune system impairment, disruption of normal liver function, folate and other nutrients malabsorption, and induction of inflammation [327].

Vitamin D is a steroid hormone with an established role in mammary gland development through the actions of its main mediator, vitamin D receptor (VDR). Through VDR, vitamin D is known to exhibit an anti-proliferative, pro-differentiating, and pro-apoptotic effect. The active form of vitamin D, 1,25(OH)2D, is responsible for the activation of VDR, therefore, circulating 25(OH)D could potentially have an inverse association with breast cancer risk [102]. Nevertheless, these results should be cautiously interpreted in the light of the consistently null associations of genetically predicted circulating 25(OH)D and breast cancer observed in Mendelian randomisation studies [328–330].

There are multiple mechanisms through which fiber uptake could have a protective role against breast cancer development [235]. It has been suggested that fiber delays gastric emptying and increases small intestine transit time, which result in reduced glucose absorption and insulin secretion. Furthermore, fiber could reduce circulating estrogens by promoting their fecal excretion during the enterohepatic circulation. Moreover, fiber seems to reduce reabsorption of estrogens through a reverse effect in intestinal β-glucuronidase activity, which is an essential step for the absorption of hydrolysed conjugated estrogens [235].

The mechanisms explaining the association of breast density with increased BrCa risk have not been clearly determined [331]. Increased breast density reflects an increased proportion of fibroglandular tissue, which could also depict an increased number of epithelial cells more susceptible to carcinogenesis and proliferation. Moreover, known determinants of breast density, such as late menopause, low parity, and use of estrogens, have been found to have a clear role on BrCa risk. Dense breast tissue is believed to exhibit a greater aromatase activity, thus resulting in hormonal sensitive tumors [49].

OC use has been found to be carcinogenic particularly when used before first childbirth. A full-term pregnancy contributes to a natural mature process of the breast epithelial cells in 2 stages, an early growth phase and a later phase of lobular differentiation. The nulliparous breast with its undifferentiated structures is more prone to the carcinogenic effects of OC use [71]. Considering estrogen-progestin therapy use, the progestin upregulates the expression of epidermal growth factor (EGF) and IGF receptors [332]. Progesterone and EGF significantly increase cell proliferation [333]. Non-steroidal anti-inflammatory drugs (NSAIDS) use appears to be protective through their effect on prostaglandin E2, which has been shown to up-regulate aromatase expression in adipose tissue fibroblasts by promoting binding of various transcription factors to aromatase promoters I.3 and II [334]. The structural similarity of digoxin and other cardiac glycosides to digitalis compounds like estradiol could explain the observed positive association with BrCa [65].

The mechanisms by which higher education level was associated with increased risk of BrCa remains unclear, although it is likely that this association is driven by other factors. One theory could be that women of a higher educational level usually have their first childbirth at a later age and, also, have fewer children. Other explanation might be that higher educational level has been associated with later menopause, higher alcohol use, and higher prevalence of hormonal treatments. Menarche at an older age shows a protective role particularly among Luminal tumors. Although there seems to be a hormonal mechanism supporting this association, evidence shows that when estrogen receptor positive (ER +) progenitor cells are exposed to estrogen, they produce paracrine signals that cause neighboring populations of ER- cells to proliferate [335].

### Strengths and weaknesses of the study

Certain limitations should be considered with respect to the findings of this umbrella review. The analysis focused on meta-analyses of observational studies missing probably the latest evidence of primary observational studies not considered yet in any evidence synthesis. However, given the large amount of included evidence it seems unlikely that single primary studies would affect the evidence grading to a modest degree. The methodological quality of the included publications was moderate as several publications failed to report or apply critical items of the AMSTAR tool, such as the comprehensive literature search, the use of publication status as an inclusion criterion, and the use of the scientific quality of the included studies for drawing conclusions. A relatively high number of meta-analyses included less than 10 primary studies; hence, the excess significance and small study effects tests could be underpowered. Furthermore, the necessary information for the calculation of the excess statistical significance test was often absent in several meta-analyses, resulting in about one-eighth of the included meta-analyses being non-evaluable, thus likely underestimating the number of convincing associations. Although we graded the certainty of evidence according to prespecified criteria, association does not equal causation, which is difficult to demonstrate in non-randomised studies. While we prioritised meta-analyses of prospective cohort studies providing adjusted estimates, most meta-analyses also included case–control designs further accommodating our evidence interpretation. While case–control studies, especially those with suboptimal designs, are more likely to be subject to epidemiological biases, we did not restrict our analyses to cohort designs to ensure maximal comprehensiveness in the included studies. While we focused our grading only on analyses of adjusted estimates, residual or unmeasured confounding may be present. Furthermore, reverse causation cannot be excluded and the retrospective studies included in certain meta-analyses may be vulnerable to recall bias. Therefore, considering these weaknesses, we advise caution in any interpretation of the results presented in our review. Nevertheless, this umbrella review provides the most comprehensive assessment of the published epidemiological literature of non-genetic factors and BrCa risk. A vast amount of data was considered, and robust methodological approaches were used to assess the evidence. Overall, although the constraints of this umbrella review probably would make our assessment somewhat more lenient, their effect on the associations supported by *convincing* evidence is expected to be trivial.

### Implications for future research

Our findings suggest some highly modifiable protective factors for BrCa. Interestingly, while diet was the most studied exposure category, associations failed to reach higher levels of evidence, indicating the methodological limitations in the field. To improve the validity of these associations future research should focus: i) on more robust study designs, such as high quality randomised controlled trials or Mendelian randomisation studies that have the potential to minimise biases common in observational epidemiological designs, and ii) on better exposure assessment techniques including objective measures and uitilising large scale omics technology to bolster our understanding of the mechanistic evidence underlying these associations. Overall, our study provides knowledge that supports the development of BrCa prevention recommendations and guidance, both at an individual level and for public health initiatives.

## Conclusions

As the incidence of BrCa increases in many countries worldwide, the identification of modifiable risk factors is imperative for health care professionals to provide both individualised and public health guidance for BrCa prevention. Our study summarised a large number of publications describing associations between non-genetic factors and BrCa risk with varying methodological quality and varying strength and validity of the associated evidence. The validity of several well-established risk factors was reaffirmed and several risk factors with potentially higher levels of evidence strength were highlighted. These results reinforce the existing guidelines and recommendations advocating for women to maintain a healthy weight, engage in regular physical activity, and adopt a nutritious, high-fiber diet to mitigate the risk of developing BrCa. Moreover, our findings underscore the importance of regular screening, particularly for high-risk groups, i.e., women over 50 years old with increased breast density, poor lifestyle, and prior use of OC, further emphasising the proactive measures that can significantly contribute to breast cancer prevention. However, it is important to note that many associations did not reach higher levels of evidence. Studies following consistent standardisation definitions and procedures could improve the quality of publications and the level of the evidence.

### Supplementary Information

Supplementary Material 1.

Supplementary Material 2.

## Data Availability

Most of the data and the list of all meta-analyses not selected for data extraction are provided in the supplementary material. The data extracted from the primary studies can be made available upon a reasonable request.

## Abbreviations

BIRADS: Breast Imaging Reporting and Data System
BMI: Body mass index
BrCa: Breast cancer
EGF: Epidermal growth factor
ER: Estrogen receptor
HER2: Human epidermal growth factor receptor 2
IGF: Insulin-like growth factor
NSAIDS: Non-steroidal anti-inflammatory drugs
OC: Oral contraceptive
PRIOR: Preferred Reporting Items for Overviews of Reviews
SHBG: Sex hormone binding globulin
VDR: Vitamin D receptor
WCRF/AICR: World Cancer Research Fund/American Institute for Cancer Research Recommendations
