# Supplementary material for: Non-genetic factors and breast cancer: an umbrella review of meta-analyses
Source: BMC Cancer. 2024 Jul 26;24:903. doi: 10.1186/s12885-024-12641-8 (PMC11282738; doi:10.1186/s12885-024-12641-8)
Supplement: Supplementary file 1 — Supplementary Material 1. [file 12885_2024_12641_MOESM1_ESM.docx]

**Non-genetic factors and breast cancer: an umbrella review of meta-analyses**

Anneza Yiallourou ^1^, Katerina Pantavou ^1^, Georgios Markozannes ^2,3^, Antonis Pilavas ^1^, Andrea Georgiou ^1^, Andria Hadjikou ^1^, Mary Economou ^1^, Neophytos Christodoulou ^4^, Konstantinos Letsos ^1^, Elina Khattab ^1^, Chrystalleni Kossyva ^1^, Maria Constantinou ^1^, Melanie Theodoridou ^1^, Daniele Piovani ^5,6^, Konstantinos Κ. Tsilidis ^2,3^, Stefanos Bonovas ^5,6^, Georgios K. Nikolopoulos ^1^

^1^ Medical School, University of Cyprus, Nicosia 1678, Cyprus

^2^ Department of Epidemiology and Biostatistics, School of Public Health, Imperial College London, London SW7 2AZ, UK

^3^ Department of Hygiene and Epidemiology, University of Ioannina School of Medicine, Ioannina 45110, Greece

^4^ Royal Papworth Hospital, Papworth Rd, Trumpington, Cambridge, CB2 0AY, UK

^5^ Department of Biomedical Sciences, Humanitas University, Milan 20072, Italy

^6^ IRCCS Humanitas Research Hospital, Milan 20089, Italy

Correspondence: G. K. Nikolopoulos, Μedical School, University of Cyprus, Cyprus, nikolopoulos.georgios@ucy.ac.cy

**Supplemental Material**

**Table of contents**

[Supplemental Methods 3](#_Toc167731824)

[PRIOR Checklist 3](#_Toc167731825)

[Search strategy 5](#_Toc167731826)

[Exclusion criteria 6](#_Toc167731827)

[Supplemental Results 7](#_Toc167731828)

[**Description of the evidence base from the meta-analyses with crude summary effect estimates** 7](#_Toc167731829)

[**Evidence for non-genetic** **factors and breast cancer risk based on meta-analyses with fully-adjusted estimates** 8](#_Toc167731830)

[*Anthropometric measurements* 8](#_Toc167731831)

[*Biomarkers* 9](#_Toc167731832)

[*Breast characteristics* 11](#_Toc167731833)

[*Diet and dietary supplements* 12](#_Toc167731834)

[*Environment* 15](#_Toc167731835)

[*Exogenous hormones* 16](#_Toc167731836)

[*Lifestyle and social factors* 17](#_Toc167731837)

[*Medical history* 18](#_Toc167731838)

[*Medication* 19](#_Toc167731839)

[*Reproductive history and pregnancy* 20](#_Toc167731840)

[References 22](#_Toc167731841)

# **Supplemental Methods**

# **PRIOR Checklist**

(Gates M, Gates A, Pieper D, et al. Reporting guideline for overviews of reviews of healthcare interventions: development of the PRIOR statement. *BMJ* 2022;378:e070849. doi:10.1136/bmj-2022-070849.)

| **Section**  Topic | **#** | **Item** | **Location reported** | |
| --- | --- | --- | --- | --- |
| **TITLE** | | |  | |
| Title | 1 | Identify the report as an overview of reviews. | Title | |
| **ABSTRACT** | | |  | |
| Abstract | 2 | Provide a comprehensive and accurate summary of the purpose, methods, and results of the overview of reviews. | Abstract | |
| **INTRODUCTION** | | |  | |
| Rationale | 3 | Describe the rationale for conducting the overview of reviews in the context of existing knowledge. | Introduction, last paragraph | |
| Objectives | 4 | Provide an explicit statement of the objective(s) or question(s) addressed by the overview of reviews. | Introduction, last paragraph | |
| **METHODS** | | |  | |
| Eligibility criteria | 5a | Specify the inclusion and exclusion criteria for the overview of reviews. If supplemental primary studies were included, this should be stated, with a rationale. | Methods/ Eligibility criteria | |
|  | 5b | Specify the definition of ‘systematic review’ as used in the inclusion criteria for the overview of reviews. | Methods/ Eligibility criteria | |
| Information sources | 6 | Specify all databases, registers, websites, organizations, reference lists, and other sources searched or consulted to identify systematic reviews and supplemental primary studies (if included). Specify the date when each source was last searched or consulted. | Methods/ Search strategy | |
| Search strategy | 7 | Present the full search strategies for all databases, registers and websites, such that they could be reproduced. Describe any search filters and limits applied. | Additional file 1 – Search strategy | |
| Selection process | 8a | Describe the methods used to decide whether a systematic review or supplemental primary study (if included) met the inclusion criteria of the overview of reviews. | Methods/ Eligibility criteria | |
|  | 8b | Describe how overlap in the populations, interventions, comparators, and/or outcomes of systematic reviews was identified and managed during study selection. | Methods/ Eligibility criteria | |
| Data collection process | 9a | Describe the methods used to collect data from reports. | Methods/ Data extraction | |
|  | 9b | If applicable, describe the methods used to identify and manage primary study overlap at the level of the comparison and outcome during data collection. For each outcome, specify the method used to illustrate and/or quantify the degree of primary study overlap across systematic reviews. | Not applicable | |
|  | 9c | If applicable, specify the methods used to manage discrepant data across systematic reviews during data collection. | Not applicable | |
| Data items | 10 | List and define all variables and outcomes for which data were sought. Describe any assumptions made and/or measures taken to identify and clarify missing or unclear information. | Methods/ Data extraction | |
| Risk of bias assessment | 11a | Describe the methods used to *assess* risk of bias or methodological quality of the included systematic reviews. | Methods/ Quality assessment | |
|  | 11b | Describe the methods used to *collect* data on (from the systematic reviews) and/or *assess* the risk of bias of the primary studies included in the systematic reviews. Provide a justification for instances where flawed, incomplete, or missing assessments are identified but not re-assessed. | Methods/ Quality assessment | |
|  | 11c | Describe the methods used to *assess* the risk of bias of supplemental primary studies (if included). | Not applicable | |
| Synthesis methods | 12a | Describe the methods used to summarize or synthesize results and provide a rationale for the choice(s). | Methods/ Statistical analysis | |
|  | 12b | Describe any methods used to explore possible causes of heterogeneity among results. | Methods/ Statistical analysis | |
|  | 12c | Describe any sensitivity analyses conducted to assess the robustness of the synthesized results. | Methods/ Grading of the evidence; Results/ Table 2 | |
| Reporting bias assessment | 13 | Describe the methods used to *collect* data on (from the systematic reviews) and/or *assess* the risk of bias due to missing results in a summary or synthesis (arising from reporting biases at the levels of the systematic reviews, primary studies, and supplemental primary studies, if included). | Methods/ Grading of the evidence; Results/ Table 2 | |
| Certainty assessment | 14 | Describe the methods used to *collect* data on (from the systematic reviews) and/or *assess* certainty (or confidence) in the body of evidence for an outcome. | Methods/ Statistical analysis | |
| **RESULTS** | | |  | |
| Systematic review and supplemental primary study selection | 15a | Describe the results of the search and selection process, including the number of records screened, assessed for eligibility, and included in the overview of reviews, ideally with a flow diagram. | Results/ Literature search | |
|  | 15b | Provide a list of studies that might appear to meet the inclusion criteria, but were excluded, with the main reason for exclusion. | Additional file 2/ Table S1 - Excluded studies | |
| Characteristics of systematic reviews and supplemental primary studies | | 16 | Cite each included systematic review and supplemental primary study (if included) and present its characteristics. | Additional file 2/ Tables S2 and 3 |
| Primary study overlap | | 17 | Describe the extent of primary study overlap across the included systematic reviews. | Not applicable |
| Risk of bias in systematic reviews, primary studies, and supplemental primary studies | | 18a | Present assessments of risk of bias or methodological quality for each included systematic review. | Results/ Quality assessment and Additional file 2/ Table S4 |
|  |  | 18b | Present assessments (*collected* from systematic reviews or *assessed* anew) of the risk of bias of the primary studies included in the systematic reviews. | Not applicable |
|  |  | 18c | Present assessments of the risk of bias of supplemental primary studies (if included). | Non-included |
| Summary or synthesis of results | | 19a | For all outcomes, summarize the evidence from the systematic reviews and supplemental primary studies (if included). If meta-analyses were done, present for each the summary estimate and its precision and measures of statistical heterogeneity. If comparing groups, describe the direction of the effect. | Additional file 2/ Tables S2 and 3 |
|  |  | 19b | If meta-analyses were done, present results of all investigations of possible causes of heterogeneity. | Additional file 2/ Tables S2 and 3 |
|  |  | 19c | If meta-analyses were done, present results of all sensitivity analyses conducted to assess the robustness of synthesized results. | Additional file 2/ Tables S2 and 3 |
| Reporting biases | | 20 | Present assessments (*collected* from systematic reviews and/or *assessed* anew) of the risk of bias due to missing primary studies, analyses, or results in a summary or synthesis (arising from reporting biases at the levels of the systematic reviews, primary studies, and supplemental primary studies, if included) for each summary or synthesis assessed. | Results/ Overview of the available evidence; Additional file 2/ Table S2 |
| Certainty of evidence | | 21 | Present assessments (*collected* or *assessed* anew) of certainty (or confidence) in the body of evidence for each outcome. | Results/ Strength of epidemiological evidence; Additional file 2/ Table S2 |
| **DISCUSSION** | | | |  |
| Discussion | | 22a | Summarize the main findings, including any discrepancies in findings across the included systematic reviews and supplemental primary studies (if included). | Discussion/ Principal findings |
|  |  | 22b | Provide a general interpretation of the results in the context of other evidence. | Discussion/ Strengths and weaknesses in relation to other studies |
|  |  | 22c | Discuss any limitations of the evidence from systematic reviews, their primary studies, and supplemental primary studies (if included) included in the overview of reviews. Discuss any limitations of the overview of reviews methods used. | Discussion/ Strengths and weaknesses of the study |
|  |  | 22d | Discuss implications for practice, policy, and future research (both systematic reviews and primary research). Consider the relevance of the findings to the end users of the overview of reviews, e.g., healthcare providers, policymakers, patients, among others. | Discussion/ Implications for future research |
| **OTHER INFORMATION** | | | |  |
| Registration and protocol | | 23a | Provide registration information for the overview of reviews, including register name and registration number, or state that the overview of reviews was not registered. | Abstract and Methods |
|  |  | 23b | Indicate where the overview of reviews protocol can be accessed, or state that a protocol was not prepared. | Abstract and Methods |
|  |  | 23c | Describe and explain any amendments to information provided at registration or in the protocol. Indicate the stage of the overview of reviews at which amendments were made. | Non-applicable |
| Support | | 24 | Describe sources of financial or non-financial support for the overview of reviews, and the role of the funders or sponsors in the overview of reviews. | Funding |
| Competing interests | | 25 | Declare any competing interests of the overview of reviews' authors. | Competing interests |
| Author information | | 26a | Provide contact information for the corresponding author. | Title page |
|  |  | 26b | Describe the contributions of individual authors and identify the guarantor of the overview of reviews. | Author contributions |
| Availability of data and other materials | | 27 | Report which of the following are available, where they can be found, and under which conditions they may be accessed: template data collection forms; data collected from included systematic reviews and supplemental primary studies; analytic code; any other materials used in the overview of reviews. | Data sharing |

# **Search strategy**

**PubMed**

(("breast" [MeSH Terms] OR "breast" [All Fields] OR "breasts" [All Fields] OR "breast s" [All Fields] OR ("mammaries" [All Fields] OR "mammary glands, human" [MeSH Terms] OR ("mammary" [All Fields] AND "glands" [All Fields] AND "human" [All Fields]) OR "human mammary glands" [All Fields] OR "mammary" [All Fields] OR "breast" [MeSH Terms] OR "breast" [All Fields])) AND ("cancer*" [All Fields] OR "neoplasm*" [All Fields] OR "malignant*" [All Fields] OR "tumour*" [All Fields] OR "tumor*" [All Fields] OR "carcinoma*" [All Fields] OR "adenocarcinoma*" [All Fields]) AND ("meta analysis" [Publication Type] OR "meta analysis as topic" [MeSH Terms] OR "meta analysis" [All Fields] OR "systematic review" [All Fields] OR ("systematic review" [Publication Type] OR "systematic reviews as topic" [MeSH Terms] OR "systematic review" [All Fields]))) AND (humans [Filter])

**Scopus**

TITLE-ABS-KEY (((breast OR mammary) AND (cancer* OR neoplasm* OR malignant* OR tumour* OR tumor* OR carcinoma* OR adenocarcinoma*)) AND (meta-analysis OR "systematic review" OR systematic AND review))

**Cochrane database of systematic reviews**

(Breast OR mammary) AND (cancer* OR neoplasm* OR malignant* OR tumour* OR tumor* OR carcinoma* OR adenocarcinoma*)

# **Exclusion criteria**

**Table S1** Characteristics disqualifying systematic reviews and meta-analyses from inclusion in the umbrella review of meta-analyses on non-genetic risk factors and breast cancer

| **Exclusion criteria** |
| --- |
| Male or mix male and female breast cancer (BrCa) |
| Meta-analyses on mixed cancer without analysis exclusively on BrCa risk |
| Associations between genes or genetic markers and BrCa risk |
| Associations focusing on the survival or on prognostic factors of the survival of BrCa cases |
| Meta-analyses of clinical trials |
| Narrative reviews and reviews/ meta-analyses based on non-systematic search strategies, including non-systematic pooled analyses |
| Meta-analyses not providing effect estimates and measures of variation (or data to reproduce them) of the individual studies |
| Meta-analyses including one independent primary study |
| Meta-analyses where the comparison was made against a reference population (in which the effect metric was the standardised incidence rate) |
| Papers published in a language other than English |

# **Supplemental Results**

**Description of the evidence base from the meta-analyses** **with crude summary effect estimates**

A total of 114 meta-analytical associations (52 publications) included (either in totality or partially) primary studies with crude summary effect estimates and were subsequently excluded from the evidence grading. A brief description is provided below.

The median number of included studies in the 114 meta-analyses was 7 (range 2 to 80). One-hundred and nine (95.6 %) meta-analytic estimates pertained to overall BrCa incidence or mortality, while 5 (4.4 %) focused on BrCa receptor-related subtypes, and 2 (1.8 %) specifically to the spread into the surrounding breast tissue. Most associations (*n*= 68, 59.6 %) pertained to general population, while 6 (5.3%) associations pertained to menopausal status and 40 (35.1 %) to specific populations.

The identified non-genetic factors were classified in 11 categories (anthropometric measurements, biomarkers, breast characteristics, diet and dietary supplements, environment, exogenous hormones, family history – Consanguinity, lifestyle and social factors, medical history, medication, and reproductive history and pregnancy). Most of the 114 meta-analyses including (either in totality or partially) primary studies with crude summary effect estimates (**Additional file 2 - Table S3**) examined the association between BrCa risk and non-genetic factors classified in the category of reproductive history and pregnancy (*n*= 23, 20.2 %). Exposures classified in the category of medical history were examined by 15.8 % (*n*= 18) of the 114 meta-analyses, and in breast characteristics by 14.9 % (*n*= 17) of these meta-analyses. A high number of meta-analysis results was found also for the category of lifestyle and social factors (*n*= 14, 12.3 %).

About half (*n*= 52; 45.6 %) of the 114 meta-analyses were statistically significant (random-effects *P*-value<0.05). Of these, 5 (9.6 %) associations indicated a decreased risk for BrCa and 47 (90.4 %) an increased risk for BrCa. At a *P-*value threshold of 10^-3^, 30 (26.3 % of the 114) meta-analyses were significant (of these 30, *n*= 28 or 93.3 % indicating an increased risk). For a *P-value* threshold of 10^-6^, 19 (16.6 % of the 114) meta-analyses remained significant (of these 19, *n*= 18 or 94.7 % indicating an increased risk).

High heterogeneity (I^2^≥50%) was found in 50 (43.9 % of the 114) meta-analyses and in 22 (42.3 % of the 52) among the statistically significant (*P*-value<0.05) ones. The 95% prediction intervals excluded the null value (i.e., 1 for binary outcomes) in 22 (19.3 % of the 114) associations. Evidence of small study effects was observed in 13 (11.4 % of the 114) meta-analyses. Evidence of excess significance bias was observed in 9 (8 % of the 114) meta-analyses. For 37 (32.5 % of the 114) meta-analyses, excess significance bias was not estimated because information was missing for at least 20% of the primary studies.

**Evidence for non-genetic** **factors and breast cancer risk based on meta-analyses with fully-adjusted estimates**

*Anthropometric measurements*

**Anthropometry:** A higher body mass index (BMI) was positively associated with postmenopausal BrCa mortality [1] [Odds Ratio (OR): 1.33; 95% Confidence Interval (CI): 1.20-1.48], but not with premenopausal BrCa mortality [1]. When a dose-response pattern (5 kg/m^2^ increase) was considered, the association between BrCa risk and BMI was in opposite directions among post- [Risk Ratio (RR): 1.13; 95%CI: 1.10−1.15; Grade: Highly suggestive] and premenopausal women (RR: 0.94; 95%CI: 0.91−0.98) [2]. A dose-response (5 kg/m^2^ increase) association was observed also among postmenopausal women in the subgroup analyses for estrogen receptor-positive (ER+; RR: 1.19; 95%CI: 1.12−1.26; Grade: Highly suggestive), progesterone receptor-positive (PR+; RR: 1.47; 95%CI: 1.36−1.59; Grade: Convincing) and ER+PR+ (RR: 1.25; 95%CI: 1.19−1.32; Grade: Highly suggestive) BrCa [2].

Body weight (high vs low) and height (per 10cm increment) were both associated with BrCa. Body weight was linked to ER+ PR+ (RR: 1.82; 95%CI: 1.55−2.15; Grade: Highly suggestive) and ER- PR+ (RR: 2.01; 95%CI: 1.22−3.31) BrCa among post-, and to ER+ PR+ BrCa (RR: 0.80; 95%CI: 0.70−0.92) among premenopausal women [3]. Obesity (RR: 1.22; 95%CI: 1.13−1.32) [4] and overweight (RR: 1.15; 95%CI: 1.09−1.22) [4] were associated with increased risk of BrCa among postmenopausal women. Adult height was found to be a risk factor for BrCa (RR: 1.17; 95%CI: 1.15−1.20; Grade: Highly suggestive) [5].

The association between central obesity and BrCa was assessed in terms of waist-to-hip ratio (WHR) and waist circumference (WC). Both were risk factors in premenopausal women (RR: 1.14; 95%CI: 1.001−1.29 and RR: 1.15; 95% CI: 1.05−1.26 respectively; per 0.1 unit increment) while WC was a risk factor in postmenopausal women (RR: 1.07; 95%CI: 1.02−1.13) [2]. Individuals with increased fat mass (RR: 1.41; 95% CI: 1.21-1.65; highest vs lowest percent) [6] had a higher risk for BrCa.

**Anthropometry, early life:** Perinatal factors have been examined in association with BrCa due to the in-utero endogenous estrogen exposure. Birth length (RR: 1.31; 95%CI: 1.10−1.57) [7] and birth weight (RR: 1.11; 95%CI: 1.00−1.23 [8], RR: 1.02; 95%CI: 1.00−1.03; per 500 g increment [9], and OR: 1.07; 95% CI: 1.02−1.12; per 1 kg increment [10]) were found to increase the risk for BrCa. A higher BMI (per 5 kg/m^2^ increment) in ages 18−30 years was inversely associated with both pre- (RR: 0.86; 95%CI: 0.78−0.95) and postmenopausal (RR: 0.80; 95%CI: 0.75−0.87; Grade: Highly suggestive) BrCa risk [2]. Finally, childhood BMI was not associated with BrCa mortality [11].

**Weight fluctuation.** Four meta-analyses investigated the association between weight gain and BrCa risk [2,12–14]. Adult weight gain increased the risk of ER+PR+ (RR: 1.91; 95%CI: 1.50−2.42; high vs low) and ER-PR- tumors (RR: 1.28; 95%CI: 1.03−1.60; high vs low) [13], particularly among postmenopausal women (ER+PR+, RR: 1.12; 95%CI: 1.06−1.17 and ER-PR-, RR: 1.02; 95%CI: 1.00−1.05; per 5 kg/m^2^ increment) [2]. Moreover, adult weight gain increased BrCa risk in postmenopausal women (RR: 1.54; 95%CI: 1.39−1.72; high vs low and RR: 1.07; 95%CI: 1.06−1.09; per 5 kg/m^2^ increment; Grade: Highly suggestive) [2,14], and in the postmenopausal groups of hormone replacement therapy (HRT) non-users (RR: 1.11; 95%CI: 1.08−1.13; per 5 kg/m^2^ increment; Grade: Convincing) [12] and in never/former users of Post-menopausal hormone therapy (RR: 1.10; 95%CI: 1.07−1.12; high vs low; Grade: Highly suggestive) [2]. Accordingly, postmenopausal women had an increased risk of BrCa with BMI gain in adulthood (RR: 1.17; 95%CI: 1.11−1.23; per 5 kg/m^2^ increment; Grade: Convincing) [2] and a reduced risk at any weight loss compared with stable weight during adulthood (RR: 0.90; 95%CI: 0.81−1.00) [2]. There was no evidence of association between weight or BMI increase in premenopausal women [2,14]. Premenopausal women losing weight compared with stable weight during adulthood were found to be protected from BrCa (RR: 0.85; 95%CI: 0.74−0.99) [2].

*Biomarkers*

**Diabetes-related biomarkers.** Elevated levels of C-peptide (fasting/non-fasting) (RR: 1.40; 95%CI: 1.03−1.92) [15], C-peptide/insulin (RR: 1.35; 95%CI: 1.01−1.81) [16], and fasting glucose (RR: 1.12; 95%CI: 1.02−1.23; women without diabetes) [17] increased the risk of BrCa. Non-significant associations were found between fasting insulin levels and BrCa [15].

**Inflammatory biomarkers and hormones.** Circulating adiponectin levels (OR: 0.74; 95%CI: 0.60−0.91; highest vs lowest category and OR: 0.84; 95%CI: 0.75−0.95; per 5ng/mL increment) [18] were associated with decreased risk of BrCa in contrast to the circulating resistin (OR: 1.66; 95%CI: 0.98−2.81; highest vs lowest category and OR: 1.09; 95%CI: 1.01−1.18; per 1ng/mL increment) [18], the C-reactive protein levels (OR: 1.16; 95%CI: 1.06−1.27) [19], androstenedione (RR: 1.76; 95%CI: 1.45−2.14; high vs low; Grade: Convincing) [20], estradiol (RR: 1.85; 95%CI: 1.55−2.22; Grade: Highly suggestive; among postmenopausal women RR: 2.14; 95%CI: 1.86−2.46; Grade: Convincing) [20], estrone (RR: 2.07; 95%CI: 1.68−2.55; Grade Convincing), testosterone (RR:1.67; 95%CI: 1.45−1.93; Grade: Convincing), and free testosterone (RR:1.86; 95%CI: 1.56−2.21; Grade: Convincing) [20], that were associated with increased risk of BrCa. The highest category of serum levels of tumor necrosis factor-a (TNFa) were found to reduce the risk of BrCa (OR: 0.75; 95%CI: 0.58−0.97), without any evidence of a dose-response association (per 1 g/mL increment) [18]. Circulating leptin and interleukin-6 were not associated with BrCa risk [18].

The risk of BrCa increased by doubling the circulating endogenous premenopausal E2 estrogen levels (OR: 1.13; 95%CI: 1.00−1.28) [21], by higher circulating insulin-like growth factor-I (IGF-I) levels in postmenopausal women (OR: 1.30; 95%CI: 1.13−1.49) [22], and the presence of antithyroid antibodies (OR: 2.35; 95%CI: 1.62−3.39) [23]. Higher levels of sex hormone binding globulin (SHBG) in postmenopausal women had a protective association with BrCa risk (RR: 0.61; 95%CI: 0.52−0.71; Grade: Convincing) [24].

No association was found between urinary 6-sulfatoxymelatonin (aMT6s) [25], serum total bilirubin [26], gonadotropin [27], chorionic gonadotropin [27] and BrCa risk .

**Lipoproteins and fatty acids.** Total cholesterol (high vs low) [28], low-density lipoprotein cholesterol levels [28], and high-density lipoprotein cholesterol levels (per 1 mmol/l increment) [29] were not related to the risk of BrCa. However, a marginal dose-dependent association was found between total cholesterol (HR: 0.97; 95%CI: 0.94−1.00; per 1 mmol/L increment) [29] and BrCa risk. Moreover, high levels of high-density lipoprotein cholesterol were associated with decreased risk of BrCa (RR: 0.85; 95%CI: 0.72, 0.99) [28], when compared with low levels.

Total saturated fatty acids, palmitoleic and stearic acids, were found to be unconnected to BrCa risk. Only the palmitic acid was found to be associated with increased risk of BrCa in postmenopausal women (RR: 1.89; 95%CI: 1.20−2.98) [30]. The monounsaturated fatty acids (RR: 2.27; 95%CI: 1.07−4.81) [30] were also found to increase the risk of BrCa in postmenopausal women. Long-chain n-3 polyunsaturated fatty acid (PUFA) were found to reduce the risk of BrCa (RR: 0.67; 95%CI: 0.53−0.86) as well as those located in erythrocytes (RR: 0.19; 95%CI: 0.13−0.27), while there was no association for long-chain n-3 PUFA located in adipose tissue and BrCa risk [31]. More specifically, a protective association with BrCa was found for the ratio of n-3/n-6 PUFA (RR: 0.66; 95%CI: 0.52−0.82 in phospholipids and RR: 0.90; 95%CI: 0.82−0.99 in serum or diet) [31,32], the docosahexaenoic (RR: 0.64; 95%CI: 0.48−0.84) [31], the docosapentaenoic (RR: 0.51; 95%CI: 0.34−0.76) [31], eicosapentaenoic acids (RR: 0.69; 95%CI: 0.58−0.81) [31]. Total trans [33], n-3 [34] and n-6 [35] PUFA, alpha linolenic acid [30], linoleic acid [36], oleic acid [30], arachidonic acid [31] and triglycerides [28] and apolipoprotein A [28] were not associated with BrCa risk.

**Micronutrients.** Plasma total carotenoids (RR: 0.74; 95%CI: 0.57−0.96; highest vs lowest category and RR: 0.78; 95%CI: 0.62−0.997; per 100 μg/dl increment) [37], a-carotene levels (RR: 0.79; 95%CI: 0.66−0.94; highest vs lowest and RR: 0.82; 95%CI: 0.72−0.93; per 10 μg/dl increment) [37], b-carotene levels (RR: 0.74; 95%CI: 0.56−0.995; per 50 μg/dl increment) [37], lutein levels (RR: 0.70; 95%CI: 0.52−0.96; highest vs lowest category and RR: 0.68; 95%CI: 0.52−0.90; per 25 μg/dl increment), lutein/zeaxanthin levels (RR: 0.76; 95%CI: 0.59−0.98; highest vs lowest and RR: 0.68: 95%CI: 0.48−0.97; per 25 μg/dl increment), and lycopene levels (RR: 0.85 95%CI: 0.74−0.98 per 25 μg/dl) [37] were inversely associated with BrCa risk. The effect estimates of plasma b-carotene (highest vs lowest category), b–cryptoxanthin and zeaxanthin levels (highest vs lowest category and per 5 μg/dl) [37], and retinol (Vitamin A) [38] were statistically non-significant.

Pyridoxal 5’-phosphate (the active form of vitamin B6) serum levels were found to protect from BrCa (RR: 0.80; 95%CI: 0.66−0.98) [39]. Vitamin D [25(OH)D] was also found to have a protective role in BrCa for the highest vs lowest levels comparison (OR: 0.64; 95%CI: 0.56−0.73) [40], the deficient vs non-deficient comparisons (<10 ng/mL vs ≥10 ng/mL; OR: 2.47; 95%CI: 1.33−4.58) [41], and in the per 5 nmol/l (ΟR: 0.94; 95%CI: 0.93−0.96; Grade: Highly suggestive) [40] dose-response analysis. Other vitamins, such as pyridoxine (vitamin B6) [42], cobalamin (vitamin B12) [42], and a-tocopherol (vitamin E) [38] were not associated with BrCa.

The meta-analyses of serum calcium (RR: 0.80; 95%CI: 0.66−0.97; per 1mmol/L increment) [43] and iron (RR: 1.22; 95%CI: 1.01−1.47) [44] showed a significant association with the risk of BrCa. Cadmium [45], folate [46,47], serum ferritin [44], and transferrin saturation [44] were not associated with BrCa risk.

The phytoestrogens daidzein (OR: 0.66; 95%CI: 0.47−0.92; highest vs the lowest quantile) [48] and genistein (OR: 0.72; 95%CI: 0.54−0.96; highest vs the lowest quantile) [48] were associated with BrCa but not the equol [48] and enterolactone [49].

Urinary phthalate metabolite levels were not associated with BrCa but the subgroup analysis of urinary di(2-ethylhexyl) phthalate [DEHP] metabolite level (OR: 1.24 (1.04−1.48) and the levels of mono-n-butyl phthalate/ mono-iso-butyl phthalate (MBP/MiBP) family of metabolites (OR: 0.76; 95%CI: 0.58−0.98) [50] produced significant pooled effect estimates. The pre-diagnostic derivatives of reactive oxygen metabolites (d-ROM) were found to be a risk factor for BrCa (HR: 1.45; 95%CI: 1.01−2.09) [51].

*Breast characteristics*

Several meta-analyses investigated mammographic density [52–56] as a marker of BrCa risk using qualitative [i.e., Breast Imaging Reporting and Data System (BIRADS) or Wolfe classifications] and quantitative (e.g., percent density) measures. There was significant evidence of an increased risk of BrCa in women with BIRADS B (OR: 1.74; 95%CI: 1.46−2.07), C (OR: 1.97; 95%CI: 1.58−2.44) and D (D vs A OR: 2.14; 95%CI: 1.62−2.83; D vs B OR: 2.11; 95%CI: 1.84−2.42; Grade: Convincing) [56,57]. An increasing trend of BrCa risk with Wolfe grade was found for P1 (RR: 1.76; 95%CI: 1.41−2.19; Grade: Highly suggestive), P2 (RR: 3.05; 95%CI: 2.54−3.66; Grade: Highly suggestive), and Dy (RR: 3.98; 95%CI: 2.53−6.27; Grade: Highly suggestive) grades compared to N1. This was consistent with the findings of dichotomized Wolfe grade [P2+Dy versus (vs) N1+P1; RR: 1.86; 95%CI: 1.49−2.32) [52]. Western women with a higher percent density (i.e., high and medium vs minimal) had increased risk of ER+ (RR: 3.05; 95%CI: 2.20−4.24; Grade: Highly suggestive and RR: 2.43; 95%CI: 2.12−2.78; Grade: Convincing, respectively) and ER- BrCa (RR: 1.95; 95%CI: 1.40−2.73 and RR: 3.15; 95%CI: 1.68−5.93, respectively) [52]. Low (vs minimal) percent density was associated to ER+ BrCa (RR: 1.38; 95%CI: 1.24−1.54; Grade: Convincing) [52]. Menopausal status was examined in a meta-analysis in Asian women [53] identifying an association of percent density (high vs low) with BrCa in both pre- (OR: 3.22; 95%CI: 2.23−4.66) and postmenopausal women (OR: 1.62; 1.13−2.32).

*Diet and dietary supplements*

**Alcohol consumption.** Alcohol drinking (RR: 1.09; 95%CI: 1.06–1.12; current vs non-drinkers; Grade: Convincing [4], and RR:1.09; 95%CI: 1.06–1.12; ≤1 drink/day vs non-drinking [57], and RR: 1.22; 95%CI: 1.07–1.39; former vs non-drinkers [4]) and wine drinking (RR: 1.36; 95%CI: 1.20–1.54) [58] were risk factors of BrCa. When the analysis for alcohol consumption was stratified by tumor type, there was an association between alcohol consumption and both ER+ PR+ (RR: 1.22; 95%CI: 1.11–1.34) and ER+ PR- tumor (RR: 1.28; 95%CI: 1.07–1.53) BrCa, while there was no association for ER- PR- tumors [59].

**Carbohydrates.** An increase in BrCa risk was found for the glycemic index (RR: 1.05; 95%CI: 1.01−1.08; high vs low [60] and RR: 1.04; 95%CI: 1.00−1.07; per 10 units/day [61]) and specifically in postmenopausal women (RR: 1.06; 95%CI: 1.02−1.10; per 10 units/day) [61]. There was no evidence of an association of the risk of BrCa with carbohydrate intake [61,62], glycemic load [60,61] or sugar [62].

**Coffee, tea and beverages.** The consumption of sugar-sweetened beverages was associated with an increased risk of BrCa among premenopausal women (OR: 1.36; 95%CI: 1.05−1.76; higher vs lower consumption) [63]. Tea was found to be protective against BrCa (OR: 0.79; 95%CI: 0.65−0.95) [64]. Coffee or decaffeinated coffee intake [65] and green tea [66] did not reach statistical significance.

**Dairy products.** A higher intake of total dairy food (RR: 0.85; 95%CI: 0.76−0.95) [67], cheese (RR: 0.95; 95%CI: 0.91−1.00; per 30g/day increase) [68], skim milk (RR: 0.96; 95%CI: 0.92−1.00; per 200g/day increase) [69], and dairy or milk consumption (OR: 0.75; 95%CI: 0.62−0.89) [70] were inversely associated with BrCa. Nevertheless, yogurt consumption [68,69] had no association with BrCa risk.

**Dietary patterns.** Adherence to a prudent dietary pattern (fruit, vegetables, poultry, fish, low-fat dairy, and whole grains) and Mediterranean diet protected from BrCa (RR: 0.82; 95%CI: 0.75−0.89 [71] and RR: 0.92; 95%CI: 0.89−0.96 [72], respectively). The effect size was similar among premenopausal women (RR: 0.77; 95%CI: 0.61−0.98), ER+ and/or PR+ (RR: 0.80; 95%CI: 0.65−0.97), ER- and/or PR- BrCa (RR: 0.68; 95%CI: 0.56−0.83) for the prudent pattern [71] and among postmenopausal (RR: 0.92; 95%CI: 0.86−0.99), ER+/PR+ (RR: 0.95; 95%CI: 0.90−1.00), and ER-/PR+ BrCa (RR: 0.71; 95%CI: 0.56−0.88) for the Mediterranean diet [72]. The Western pattern (red and/ or processed meats, refined grains, potatoes, sweets, and high-fat dairy) was associated with increased risk of BrCa (RR: 1.14; 95%CI: 1.02−1.28) [71]. This association was also significant among postmenopausal women (RR: 1.19; 95%CI: 1.06−1.35) and for ER+ and/or PR+ BrCa (RR: 1.17; 95%CI: 1.04−1.33) [71]. An increase in BrCa risk was found for the adherence to the drinker dietary pattern (OR: 1.21; 95%CI: 1.04−1.41) [73], for elevated dietary inflammatory index (RR: 1.32; 95%CI: 1.17−1.48) [74], and specifically among premenopausal women (RR: 1.50; 95%CI: 1.10−2.05) [74], as well as for women exposed to early-life energy restriction (i.e., between 220 and 1660kcal/day; RR: 1.28; 95%CI: 1.04−1.57) [75]. There was no evidence of an association of the risk for BrCa with pesco vegeterian [76], semi vegetarian [77], vegetarian [76] and vegetarian/vegan diets [78].

**Fats and lipids.** Cholesterol intake was associated with 29% higher BrCa risk (RR: 1.29; 95%CI: 1.06−1.56) [79]; an association that was statistically significant only among premenopausal women (RR: 1.24; 95%CI: 1.05−1.47) [79]. The risk of BrCa was also associated with dietary total fat (RR: 1.10; 95%CI: 1.02−1.19) [34], while marine n-3 PUFA prevented BrCa (RR: 0.85; 95%CI: 0.76− 0.96) [33]. The linoleic [35] and the conjugated linoleic acid (predominant n-6 fatty acid) [80], total n-3 PUFA [80], the ratio of n-3/n-6 PUFA [81], the saturated [82], monosaturated, and polyunsaturated fat [34], and the trans fat [83] had no association with the risk of BrCa.

**Fish, meat, and animal products.** Processed meat consumption was associated with increased risk of BrCa [RR: 1.06; 95%CI: 1.02−1.11; high vs low [84] and Hazard Ratio (HR): 1.18; 95%CI: 1.04−1.33; per 50g/d increase [68]]. Similarly, red meat (RR: 1.05; 95%CI: 1.00−1.11; high vs low [4] and HR: 1.10; 95%CI: 1.03−1.18; per 100g/d increase [68]) and total red meat (HR: 1.07; 95%CI: 1.01−1.13; per 100g/d increase [68] and RR: 1.27; 95%CI: 1.12−1.44; among premenopausal women [85]) increase the risk for BrCa. There was no association for fish [68,86], egg [68,87,88], and poultry [68]. Unprocessed red meat including two comparisons based on N-acetyltransferase 2 (*NAT2*) acetylator genotype (per 25 g/day), i.e., for women characterized as NAT2 rapid or slow acetylator genotypes [89], was not associated with BrCa.

**Nutrients and vitamins.** Evidence of a dose response association (per 300 mg/day increment) suggested that exposure to calcium, as dietary (RR: 0.97; 95%CI: 0.95−0.98) [90] and total intake (i.e., dietary and/or supplemental; (RR: 0.98; 95%CI: 0.96−0.99) [90], reduced the risk of BrCa. The protective effect of total calcium intake was detected among pre- (RR: 0.92; 95%CI: 0.87−0.98) and postmenopausal (RR: 0.98; 95%CI: 0.97−0.99) [90] women as well as when a high vs low exposure was examined (RR: 0.92; 95%CI: 0.86−0.99) [90]. Similarly, total selenium (OR: 0.88; 95%CI: 0.80−0.96) [91], folate (OR: 0.98; 95%CI: 0.97−0.99; per 100 mg/day increase [45]), dietary folate (OR: 0.84; 95%CI: 0.77−0.91 [46]), and methionine intake (RR: 0.94; 95%CI: 0.89−0.99) [38] protected from BrCa. A marginally significant association was found between acrylamide and BrCa among postmenopausal women (RR: 0.93; 95%CI: 0.87−1.00) [92]. On the other hand, dietary heme iron intake (RR: 1.12; 95%CI: 1.04−1.22) [43] was associated with an increased risk of BrCa. No associations were found for dietary, supplemental, or total iron intake [43], as well as for cadmium intake [44].

A variety of plant and animal based types of vitamin A were examined as risk factors for BrCa in three meta-analyses [36,93,94]. Intake of total vitamin A (OR: 0.82; 95%CI: 0.74−0.91) [93] and total and dietary b-carotene (OR: 0.82; 95% CI 0.75−0.89 [94], RR: 0.96; 95 % CI: 0.92−1.00 per 5000 μg/day [36] and RR: 0.75; 95 % CI: 0.58−0.97 per 50 μg/day increase) [36] were associated with reduced BrCa risk. Dietary a-carotene had a protective role in BrCa (OR: 0.88 95%CI: 0.81−0.95) [94]; however, no dose-response evidence (per 1000 μg/day and 10 μg/day) [36] was detected. Intake of total carotenoids (per 10000 μg/day) [36] and dietary/supplemental b-carotene [94], and dietary beta-cryptoxanthin (highest vs lowest and per 150μg/day), lutein/+zeaxanthin and lycopene (highest vs lowest and per 10000 μg/day increase) [36] intake had no association with BrCa. Among vitamins B2 [95], B6 [38], B12 [38], C [96], and D [39,97] (dietary, supplemental and total) examined in meta-analyses, only B2 (RR: 0.90; 95%CI: 0.82−0.99) [41] and C (RR: 0.96; 95%CI: 0.81−0.92) [96] were associated with BrCa. Supplemental multivitamin intake and BrCa risk was examined in one meta-analysis producing non-significant pooled effect estimates [98].

**Plant-based food and their compounds.** Total fiber (RR: 0.95; 95%CI: 0.91−0.98 per 10gr/day increase [99] and among postmenopausal women RR: 0.91; 95% CI: 0.88−0.95; High vs low; Grade: Convincing [101]), fruit fiber (RR: 0.93; 95%CI: 0.89−0.97; high vs low) [100], vegetable fiber (RR: 0.95; 95%CI: 0.91−0.99; high vs low) [101], and soluble fiber intake (RR: 0.89; 95%CI: 0.83−0.96; high vs low) [99,100] were protective against BrCa, although the comparisons for insoluble fiber intake (RR: 0.92; 95%CI: 0.85−1.00) [101] was marginally significant. The associations for cereal and legume fiber intake were non-significant [100,101]. Flavonoid intake was examined for individual classes (i.e., anthocyanidins, flavones, flavonols, flavanones, flavan-3-ols, isoflavones) [102,103] and isoflavone-rich foods [104]. Significant protective associations were found for flavonols (OR: 0.85; 95%CI: 0.76−0.96) [103], flavan-3-ols (OR: 0.94; 95%CI: 0.89−0.98) [102], and isoflavone intake (OR: 0.71; 95%CI: 0.64−0.78) [102]. A protective association was also found for the consumption of fruits and vegetables (RR: 0.89; 95%CI: 0.80−0.99; high vs low [105] and ΗR: 0.98; 95%CI: 0.97−1.00; per 100 g/day increase) [69], as well as individually for fruits (RR: 0.92; 95%CI: 0.86−0.98; high vs low [105], ΗR: 0.97; 95%CI: 0.95−0.99; per 100 g/day increase [69] and RR: 0.94; 95%CI: 0.89−1.00; per 200 g/day increase [105]) and citrus fruits (OR: 0.90; 95%CI: 0.85−0.96) [106]. Fruit juice was not associated with BrCa [64,69]. Carrot (OR: 0.81; 95%CI: 0.70−0.93) [107], cruciferous vegetables (OR: 0.88; 95%CI: 0.81−0.95) [108], onions (RR: 0.81; 95%CI: 0.68−0.96) [109], and mushrooms intake (RR: 0.97; 95%CI: 0.95−0.98) [110] were found to reduce BrCa risk. No association was detected between garlic [109] and nuts [69,111], and BrCa.

Soy (RR: 0.86; 95%CI: 0.80−0.94), soy food and/or soy isoflavones intake (HR: 0.97; 95%CI: 0.95−0.99; per 10 mg/day) [112], and tofu (RR: 0.78; 95%CI: 0.70−0.88) [113] were found to be protective against BrCa, while miso intake was marginally protective against BrCa (RR: 0.89; 95%CI: 0.79−1.00) [114].

Finally, olive oil (RR: 0.67; 95%CI: 0.52−0.86) [115] and whole grains (RR: 0.86; 95%CI: 0.77−0.96 [116] per 50 g/day increase) were associated with BrCa, whereas legumes [117] and lignans, matairesinol, and secoisolariciresinol [102] were not associated with BrCa.

*Environment*

**Air quality.** Ambient air quality exposure was examined through the concentrations of nitrogen dioxide (NO_2_) [118] and particulate matter (PM) [119]. An increase in each 10 μg/m^3^ in NO_2_ exposure (HR: 1.02; 95%CI: 1.01−1.04) [118] was associated with increased risk for BrCa. Neither PM_2.5_ nor PM_10_ were associated with BrCa risk [119].

**Endocrine-disrupting chemicals.** Bisphenol A and phthalates were not associated with BrCa except for mono-2-isobutyl phthalate (OR: 0.75; 95%CI: 0.58−0.98) [120] and mono-benzyl phthalate (OR: 0.74; 95%CI: 0.57−0.95) [120].

**Radiation.** Exposure to low frequency electromagnetic fields (EMF; <300 Hz) generated by power equipment, appliances or transmission lines (OR: 1.08; 95%CI: 1.00−1.16) [121] and exposure to occupational and residential EMF (RR: 1.09; 95%CI: 1.04−1.14) [122] were associated with increased risk of BrCa. A protective association was found between solar radiation and BrCa; however, only for ≥1 vs <1h/day exposure in summer months (RR: 0.84; 95%CI:0.77−0.91) [123]. No association was found between X-rays exposure and BrCa risk in an Indian population [124].

**Surroundings.** The exposure at urban residential environment (RR: 1.09; 95%CI: 1.01−1.19) [125] was associated with BrCa. Nevertheless, the association between greenspaces and BrCa was statistically non-significant.

**Toxic chemicals.** Environmental tobacco smoke exposure (i.e., passive smoking, second-hand smoke) in non-smoking women was associated with an increased risk of BrCa (RR: 1.15; 95%CI: 1.07−1.23) [126]. Significant increases in the risk of BrCa was observed among women with higher plasma/fat levels of polychlorinated biphenyl (PCB) 99 (OR: 1.35; 95%CI: 1.02−1.80) [127], 183 (OR: 1.56; 95%CI: 1.25−1.95) [127], and 187 (OR: 1.28; 95%CI: 1.04−1.57) [127]. Moreover, the risk of BrCa was associated with the potentially anti-estrogenic and immunotoxic, and dioxin-like PCBs (OR:1.23; 95%CI: 1.08−1.40; exposed vs non-exposed) [128]. Rinse users (OR: 1.17; 95%CI: 1.02−1.35) [129] and users of (any) hair dye (OR: 1.08; 95%CI: 1.01−1.16) [129] were found to have an increased risk for BrCa. No association was found regarding the exposure to dichlorodiphenyldichloro-ethylene [130], methylene chloride [131], and polyfluoroalkyl substances [132] — perfluorohexane sulfonic acid (PFHxS), perfluorononanoic acid (PFNA), perfluorooctanoic acid (PFOA), and perfluorooctane sulfonic acid (PFOS).

*Exogenous hormones*

The use of HRT (RR: 1.26; 95%CI: 1.20−1.32) [133] was associated with an increased risk of BrCa. This association varied depending on the type of HRT; the risk was greater in estrogen–progestin (HR: 1.56; 95%CI: 1.37−1.76; Grade: Highly suggestive) than in estrogen-only therapy users (HR: 1.16; 95%CI: 1.09−1.25) [134], when compared to non-users. Any hormonal therapy (RR: 1.33; 95%CI: 1.13−1.56) [135] was associated with increased risk of in-situ BrCa in postmenopausal women.

In vitro fertilization (IVF) (RR: 0.78; 95%CI: 0.65−0.94) was associated with reduced BrCa risk [136]. Fertility treatments [27], clomiphene [27], and IVF in infertile women [136] were not associated with BrCa risk.

The use of oral contraceptives (OC) was found to have a non-significant association with the risk of BrCa [4]. This association was significant among premenopausal women who had an increased risk for BrCa when using OC (OR: 1.19; 95%CI: 1.09−1.29, <50 years, parous OR: 1.29; 95% CI: 1.20−1.40; Grade: Convincing, and <50 years, parous OR: 1.44; 95% CI: 1.28−1.62; Use before first full-term pregnancy vs non-use; Grade: Highly suggestive) [137]. Moreover, the use of the levonorgestrel-releasing intrauterine system was associated with an increased risk for BrCa (OR:1.16; 95%CI: 1.06−1.28) [138]. A meta-analysis [137] focusing on case-control studies and involving premenopausal women and women younger than 50 years suggested that parous women (OR: 1.29; 95%CI: 1.20−1.40), and parous women using OC before (OR: 1.44; 95%CI: 1.28−1.62) or after (OR: 1.16; 95%CI: 1.06−1.26) a first full-term pregnancy had an increased risk of BrCa whereas non-significant associations were found for nulliparous women. The age at first use of oral contraceptives [139], the presence of *BRCA1* or *BRCA2* mutations [140], and the use of progesterone [4,27] were not associated with BrCa.

*Lifestyle and social factors*

**Physical activity (PA).** Low (OR: 0.79; 95%CI:0.72–0.86) [141], high (OR:0.73; 95%CI: 0.65–0.81) [141], lifetime PA (RR:0.79; 95%CI: 0.72–0.86) [142], and PA at a young age (i.e., childhood, adolescence, and young adulthood/ age 5 to 30 years; OR: 0.81; 95%CI: 0.76–0.87) [141] were found to have a protective role in BrCa. Furthermore, women who engaged in 11.25 met-hours/week of PA (leisure time PA RR:0.93; 95%CI: 0.88–0.98) [143] had a reduced risk. The effect size for lifetime, at young age and moderate-vigorous physical exercise was similar in both premenopausal (RR: 0.82; 95%CI: 0.68–0.99, RR: 0.87; 95%CI: 0.78–0.96, and RR: 0.80; 95%CI: 0.73–0.87; Grade: Highly suggestive, respectively) and postmenopausal (RR: 0.69; 95%CI: 0.59–0.81, RR: 0.86; 95%CI: 0.78–0.94, and RR: 0.80; 95%CI: 0.75–0.84; Grade: Highly suggestive, respectively) BrCa risk [142,144]. No association was found between the level of walking and BrCa risk among pre- and postmenopausal women [2], and between vigorous activity and hormone receptor positive or negative BrCa, except for ER- postmenopausal BrCa (RR: 0.75; 95%CI: 0.57–0.99) [2]. Occupational PA exhibited a protective role in BrCa in postmenopausal women (RR:0.90; 95%CI: 0.85–0.96), while it had no effect in premenopausal women [2]. Sedentary behavior (OR: 1.08; 95%CI: 1.04–1.13 [145], OR: 1.08; 95%CI: 1.04–1.13; per 1-h/day increment [146] and OR: 1.10; 95%CI: 1.02–1.18 for occupational sedentary behavior [145]) was found to be a risk factor of BrCa whereas there was a marginal positive association between low sitting time and BrCa risk in postmenopausal women (RR: 1.20; 95%CI: 1.00–1.44) [2].

**Sleep quality and circadian norm.** Circadian disrupting (RR: 1.14; 95%CI: 1.05–1.24) [147] was found to be a risk factor of BrCa. Moreover, exposure at high artificial light at night (RR: 1.11; 95%CI: 1.07−1.15) [148] was associated with BrCa. This association was significant among both pre- (RR: 1.16; 95%CI: 1.05−1.28) [148] and postmenopausal women (RR: 1.07; 95%CI: 1.02−1.13) [147] as well as for both indoor (RR: 1.08; 95%CI: 1.00−1.16) [147] and outdoor environments (RR: 1.11; 95%CI: 1.07−1.16) [147]. Night shift work was also associated with an increased BrCa risk (RR: 1.11; 95%CI: 1.04−1.20) [148]. The association remained significant when different exposure durations were considered (RR: 1.13; 95%CI: 1.03−1.24; <10 years vs never, RR: 1.13; 95%CI: 1.01−1.27; ≥15 years vs never [149], RR: 1.54; 95%CI: 1.12−2.13; recent ≥15 years vs never [149], and RR: 1.54; 95%CI: 1.12−2.13; more than 5 times a week vs never [150]). For every extra year of intensive night shift work (equivalent to 300 shifts) there was a 4% increase in BrCa risk (RR: 1.04; 95%CI: 1.01–1.07) [151]. Neither short nor long sleep duration was associated with BrCa risk [152,153].

**Smoking.** Active smoking (ever vs never smoking) was associated with an increased risk for BrCa (RR: 1.09; 95%CI: 1.07–1.12; Grade: Highly suggestive) in a large meta-analysis of 71 studies [154]. The risk of BrCa was increased in individuals with either a longer duration of active smoking or a larger number of pack-years (RR: 1.01; 95%CI: 1.00–1.01) [154], which highlights the dose-response association between active smoking and BrCa.

**Social factors.** A higher socioeconomic status limited to the education and occupation (RR: 1.16; 95%CI: 1.10–1.23) [155], and higher educational level were associated with increased risk of BrCa (RR: 1.22; 95%CI: 1.14–1.30; Grade: Highly suggestive) [156]. Marital status exhibited a risk association with BrCa (OR: 1.20; 95%CI: 1.07–1.35; unmarried vs married, OR: 1.24; 95%CI: 1.05–1.45; lifelong single vs married) [157]. There was no evidence of association between widowed or divorced individuals and BrCa risk [157,158]. Women older than 18 years that experienced striking stressful life events were found to have an 11% increased risk for BrCa (RR: 1.11; 95%CI: 1.03–1.19) [159].

**Other.** Adherence to the World Cancer Research Fund/American Institute for Cancer Research recommendations for cancer prevention (2018 WCRF/AICR) [160] was examined through a score involving eight recommendations (I.e., body fatness, physical activity, consumption of whole grains/vegetables/fruit/beans, “fast foods” and other processed foods high in fat, starches, or sugars, red/processed meat, sugar-sweetened drinks, alcohol, breastfeeding). Higher scores indicate higher adherence. A higher adherence to the WCRF/AICR recommendations reduced the risk of BrCa (RR: 0.73; 95%CI: 0.65–0.82; high vs low; Grade: Highly suggestive, RR: 0.91; 95%CI: 0.88–0.94; per 1-point increment; Grade: Highly suggestive) [160].

*Medical history*

**Cardiometabolic diseases.** The risk of BrCa was increased in women with diabetes (OR: 1.20; 95%CI: 1.13−1.29; Grade: Highly suggestive) and, particularly, in women with type 2 diabetes (OR: 1.22; 95%CI: 1.07−1.40) [161]. Hypertension was also a risk factor (RR: 1.11; 95%CI: 1.05−1.19), while this risk was slightly increased in postmenopausal women (RR: 1.15; 95%CI: 1.08−1.23) [162]. An increased risk for BrCa was found for women with metabolic syndrome (RR: 1.15; 95%CI: 1.05−1.26 and RR: 1.16; 95%CI: 1.05−1.29; invasive BrCa) [163] and, in particular, with postmenopausal women (RR: 1.25; 95%CI: 1.12−1.39) [163].

**Endocrine and autoimmune diseases.** Patients with hyperthyroidism have an increased risk for BrCa (RR: 1.12; 95%CI: 1.07−1.17) [164]. This association was significant among post- (RR: 1.17; 95%CI: 1.05−1.29) but not among premenopausal women [164]. On the contrary, hypothyroidism was associated with a decreased risk of BrCa risk (RR: 0.69; 95%CI: 0.53−0.89; premenopausal women and (RR: 0.94; 95%CI: 0.90−0.99; postmenopausal women). There was no significant evidence of increased risk of BrCa in women with systemic sclerosis [165] or systematic lupus erythematosus [166].

**Genetic disorders.** Down syndrome was not associated with BrCa [167].

**Neurological and psychiatric diseases.** Migraine (RR: 0.84; 95%CI: 0.75−0.94) [168] and Huntington’s disease (RR: 0.59; 95%CI: 0.38−0.90) [167] were found to be associated with BrCa. An association was also found in women with anorexia nervosa (RR: 0.60; 95%CI: 0.50−0.74) [169]. This association sustained across parous (RR: 0.48; 95%CI: 0.36−0.62) and nulliparous women (RR: 0.74; 95%CI: 0.55−0.99) [169]. On the contrary, schizophrenia increased the risk of BrCa (RR: 1.24; 95%CI: 1.08−1.43) [167]. Parkinson’s disease [170], multiple sclerosis [167], and depression [171] were not associated with BrCa.

**Other factors**. Bone mineral density (BMD) (RR: 0.75; 95%CI: 0.60−0.93; measured at lumbar spine) [172], bariatric surgery (RR:0.50; 95%CI: 0.38−0.67) [173], and risk-reducing salpingo-oophorectomy (RR:0.63; 95%CI: 0.49−0.81; among BRCA1 mutation carriers, and RR:0.51; 95%CI: 0.34−0.75; among BRCA mutation carriers) [174] were associated with reduced risk of BrCa. Periodontal disease (RR:1.22; 95%CI: 1.06−1.40) [175] and off-hour hospital admission (weekends and nights) were associated with increased risk of BrCa (OR: 1.50; 95%CI: 1.21−1.86) [176]. No evidence of association was found for asthma, hay fever, atopy, or any allergy [177].

*Medication*

**Antidiabetic**. Dipeptidyl peptidase-4 (DPP-4) inhibitors had a protective role against BrCa among individuals with type 2 diabetes mellitus (vs active antidiabetic drugs; HR: 0.76; 95%CI: 0.60−0.96) [178]. No association was found between BrCa and insulin [179], metformin [180], and thiazolidinedione [181].

**Cardiovascular**. Beta (RR:1.20; 95%CI: 1.09−1.33) [182] and calcium channel blockers (RR:1.07; 95%CI: 1.02−1.12) [182], diuretics (RR1.06; 95%CI: 1.01−1.11) [182], digitalis (RR: 1.35; 95%CI: 1.25−1.46) [183] and digoxin use (RR: 1.30; 95%CI: 1.17−1.45; Grade: Highly suggestive) [184] were found to increase the risk of BrCa. The latter association remained significant considering the digoxin use of 3 years or more (RR: 1.25; 95%CI: 1.08−1.45) and in ER+ BrCa cases (RR: 1.33; 95%CI: 1.25−1.42; Grade: Highly suggestive) [184]. Antihypertensive drug use [185], angiotensin-converting enzyme (ACE) inhibitor/ angiotensin-receptor blocker (ARB) [182,185], angiotensin-receptor blockers (ARBs) [182], renin-angiotensin system inhibitors [182], spironolactone [186], and statins [187], were not significantly associated with BrCa risk.

**Non-steroidal anti-inflammatory drugs (NSAIDs)**. Aspirin (RR: 0.94; 95%CI: 0.91−0.97) [188] and ibuprofen use (OR: 0.69; 95%CI: 0.48−0.99) [189] reduced the risk of BrCa. Subgroup analysis showed that aspirin use could reduce the risk of ER+ (RR: 0.89; 95%CI: 0.82−0.97), PR+ (RR: 0.86; 95%CI: 0.78−0.98) and ER+ PR+ BrCa (RR: 0.91; 95%CI: 0.83−0.99) [188], as well as BrCa in postmenopausal women (RR: 0.92; 95%CI: 0.86−0.98) [188]. Comparisons of highest versus the lowest dosages, duration, and frequency of aspirin intake [190] or analysis by cancer stage (localised, non-localised [191] produced non-significant effect estimates.

**Other medications.** Bisphosphonates protected against primary BrCa (RR: 0.87; 95%CI: 0.80−0.94) [192]. This association was present for use more than one year (RR range between 0.68 and 0.75) [192,193] and among postmenopausal women (RR: 0.87; 95%CI: 0.77−0.97) [193] whereas it was stronger for use exceeding one year, i.e., 1-2 years (RR: 0.69 95%CI: 0.54−0.88); 2-3 years (RR: 0.73; 95%CI: 0.55−0.97); 3-4 years (RR: 0.78; 95%CI: 0.60−1.01); >4 years (RR: 0.68; 95%CI: 0.53−0.87), or for invasive BrCa (RR: 0.77; 95%CI: 0.63−0.94) [193]. Moreover, etidronate use reduced the risk of BrCa (RR:0.87; 95%CI: 0.80−0.96) [192]. On the contrary, antibiotic use (RR:1.17; 95%CI: 1.07−1.29) [194] and antipsychotic use (RR:1.39; 95%CI: 1.11−1.73) [195] increased the risk for BrCa. There was no significant association between the use of acid-suppressive agent [196], alendronate [192,197], antidepressants and antidepressive agents [198], thyroid replacement therapy [199], erotonin-norepinephrine reuptake inhibitor [198], selective serotonin reuptake inhibitor [198], and risedronate [192], and BrCa.

*Reproductive history and pregnancy*

**Age at pregnancy.** First pregnancy at an older age (OR: 1.16; 95%CI: 1.01−1.34; ER+ PR+ BrCa) [200] and late pregnancy (OR: 1.37; 95%CI: 1.26−1.50; ≥30 vs <30 years) [4] were associated with increased BrCa risk.

**Abortion.** Induced abortion [201] and spontaneous abortion [201,202] were not associated with BrCa.

**Menarche, pregnancy, and menopause.** The late age at menarche had a protective role against ER+PR+ BrCa (RR: 0.71; 95%CI: 0.67−0.75; oldest vs youngest age category; Grade: Convincing) but not against ER-PR- BrCa [203]. The subgroup analysis by menopausal status showed that late age at menarche was protective in both pre- (RR: 0.70; 95%CI: 0.60−0.82) and postmenopausal women (RR: 0.73; 95%CI: 0.62−0.85) with ER+PR+ BrCa, and only in premenopausal women (RR: 0.78; 95%CI: 0.62−0.99) with ER-PR- BrCa [203]. The association between age at menopause and BrCa was examined in one meta-analysis [204] focused on Japanese women. It suggested that premenopausal Japanese women after the age of 50 had a higher risk of BrCa than women with menopause before the age of 50 (OR: 2.22; 95%CI: 1.56−3.14) [204].

**Non-hormonal birth control.** No association was found between tubal sterilization and BrCa risk [205].

**Parity and breastfeeding.** Parity was associated with BrCa risk (RR: 1.17; 95%CI: 1.04−1.33; nulliparous vs primiparous/multiparous) [4] and with luminal A (OR: 0.66; 95%CI: 0.56−0.78; parous vs nulliparous) [206] and B (OR: 0.69; 95%CI: 0.58−0.81; parous vs nulliparous) [206] BrCa. Exclusively breastfeeding among parous women prevented BrCa (RR: 0.73 95%CI: 0.58−0.91) [207]. Compared to never breastfeed, women who breastfed for at least 12 months had a significant decrease of BrCa risk (OR: 0.73; 95%CI: 0.66−0.81) but not women who breastfed less than 12 months [208]. Ever breastfeeding prevented ER-PR- (OR: 0.79; 95%CI: 0.71−0.87) [209], ER+PR+ (OR: 0.91; 95%CI: 0.84−0.98) [209], and triple negative BrCa (OR: 0.77; 95%CI: 0.64−0.92) [200]. There was no evidence of association with human epidermal growth factor receptor 2 (HER2) positive BrCa risk [200]. Finally, the dizygotic twin pregnancy [7] was not associated with BrCa.

**Pregnancy complications.** Women with preeclampsia had a decreased BrCa risk (RR:0.88; 95%CI: 0.83−0.93) [210]. There was no evidence of association with BrCa risk for pre-eclampsia in primiparous women [211], and gestational diabetes [212].

**Other.** An advanced paternal age was marginally associated with a higher risk for BrCa in daughters (RR: 1.08; 95%CI: 1.01−1.16) [7].

# **References**

1. Chen Y, Liu L, Zhou Q, Imam MU, Cai J, Wang Y, et al. Body mass index had different effects on premenopausal and postmenopausal breast cancer risks: a dose-response meta-analysis with 3,318,796 subjects from 31 cohort studies. BMC Public Health. BMC Public Health; 2017;17:936.

2. Chan DSM, Abar L, Cariolou M, Nanu N, Greenwood DC, Bandera E V., et al. World Cancer Research Fund International: Continuous Update Project—systematic literature review and meta-analysis of observational cohort studies on physical activity, sedentary behavior, adiposity, and weight change and breast cancer risk. Cancer Causes Control. 2019;30:1183–200.

3. Suzuki R, Orsini N, Saji S, Key TJ, Wolk A. Body weight and incidence of breast cancer defined by estrogen and progesterone receptor status--a meta-analysis. Int J Cancer. Int J Cancer; 2009;124:698–712.

4. Poorolajal J, Heidarimoghis F, Karami M, Cheraghi Z, Gohari-Ensaf F, Shahbazi F, et al. Factors for the primary prevention of breast cancer: A meta-analysis of prospective cohort studies. J Res Health Sci. 2021;21:e00520.

5. Zhang B, Shu XO, Delahanty RJ, Zeng C, Michailidou K, Bolla MK, et al. Height and breast cancer risk: Evidence from prospective studies and mendelian randomization. J Natl Cancer Inst. J Natl Cancer Inst; 2015;107:djv219.

6. Namazi N, Irandoost P, Heshmati J, Larijani B, Azadbakht L. The association between fat mass and the risk of breast cancer: A systematic review and meta-analysis. Clin Nutr. Clin Nutr; 2019;38:1496–503.

7. Xue F, Michels KB. Intrauterine factors and risk of breast cancer: a systematic review and meta-analysis of current evidence. Lancet Oncol. 2007;8:1088–100.

8. Chen C, Chen X, Wu D, Wang H, Wang C, Shen J, et al. Association of birth weight with cancer risk: a dose–response meta-analysis and Mendelian randomization study. J Cancer Res Clin Oncol. Springer Berlin Heidelberg; 2023;149:3925–35.

9. Zhou W, Chen X, Huang H, Liu S, Xie A, Lan L. Birth Weight and Incidence of Breast Cancer: Dose-Response Meta-analysis of Prospective Studies. Clin Breast Cancer. Elsevier Inc.; 2020;20:e555–68.

10. Xu X, Dailey AB, Peoples-Sheps M, Talbott EO, Li N, Roth J. Birth weight as a risk factor for breast cancer: a meta-analysis of 18 epidemiological studies. J Womens Health (Larchmt). J Womens Health (Larchmt); 2009;18:1169–78.

11. Simmonds M, Burch J, Llewellyn A, Griffiths C, Yang H, Owen C, et al. The use of measures of obesity in childhood for predicting obesity and the development of obesity-related diseases in adulthood: A systematic review and meta-analysis. Health Technol Assess (Rockv). 2015;19:1–336.

12. Keum N, Greenwood DC, Lee DH, Kim R, Aune D, Ju W, et al. Adult weight gain and adiposity-related cancers: A dose-response meta-analysis of prospective observational studies. JNCI J Natl Cancer Inst. Oxford Academic; 2015;107:88.

13. Vrieling A, Buck K, Kaaks R, Chang-Claude J. Adult weight gain in relation to breast cancer risk by estrogen and progesterone receptor status: a meta-analysis. Breast Cancer Res Treat. Breast Cancer Res Treat; 2010;123:641–9.

14. Hao Y, Jiang M, Miao Y, Li X, Hou C, Zhang X, et al. Effect of long-term weight gain on the risk of breast cancer across women’s whole adulthood as well as hormone-changed menopause stages: A systematic review and dose–response meta-analysis. Obes Res Clin Pract. 2021;15:439–48.

15. Hernandez A V., Guarnizo M, Miranda Y, Pasupuleti V, Deshpande A, Paico S, et al. Association between insulin resistance and breast carcinoma: A systematic review and meta-analysis. PLoS One. 2014;9:e99317.

16. Pisani P. Hyper-insulinaemia and cancer, meta-analyses of epidemiological studies. Arch Physiol Biochem. 2008;114:63–70.

17. Boyle P, Koechlin A, Pizot C, Boniol M, Robertson C, Mullie P, et al. Blood glucose concentrations and breast cancer risk in women without diabetes: A meta-analysis. Eur J Nutr. 2013;52:1533–40.

18. Yoon YS, Kwon AR, Lee YK, Oh SW. Circulating adipokines and risk of obesity related cancers: A systematic review and meta-analysis. Obes Res Clin Pract. Asia Oceania Assoc. for the Study of Obesity; 2019;13:329–39.

19. Guo L, Liu S, Zhang S, Chen Q, Zhang M, Quan P, et al. C-reactive protein and risk of breast cancer: A systematic review and meta-analysis. Sci Rep. Nature Publishing Group; 2015;5:1–8.

20. Drummond AE, Swain CTV, Brown KA, Dixon-Suen SC, Boing L, Van Roekel EH, et al. Linking Physical Activity to Breast Cancer via Sex Steroid Hormones, Part 2: The Effect of Sex Steroid Hormones on Breast Cancer Risk. Cancer Epidemiol Biomarkers Prev. 2022;31:28–37.

21. Walker K, Bratton DJ, Frost C. Premenopausal endogenous oestrogen levels and breast cancer risk: A meta-analysis. Br J Cancer. Nature Publishing Group; 2011;105:1451–7.

22. EHaBCCG, Key TJ, Appleby PN, Reeves GK, Roddam AW, Helzlsouer KJ, et al. Insulin-like growth factor 1 (IGF1), IGF binding protein 3 (IGFBP3), and breast cancer risk: Pooled individual data analysis of 17 prospective studies. Lancet Oncol. Elsevier Ltd; 2010;11:530–42.

23. Hardefeldt PJ, Eslick GD, Edirimanne S. Benign thyroid disease is associated with breast cancer: A meta-analysis. Breast Cancer Res Treat. 2012;133:1169–77.

24. He XY, Liao YD, Yu S, Zhang Y, Wang R. Sex hormone binding globulin and risk of breast cancer in postmenopausal women: A meta-analysis of prospective studies. Horm Metab Res. 2015;47:485–90.

25. Xu J, Huang L, Sun GP. Urinary 6-sulfatoxymelatonin level and breast cancer risk: Systematic review and meta-analysis. Sci Rep. Nature Publishing Group; 2017;7:5353.

26. Monroy‐iglesias MJ, Moss C, Beckmann K, Hammar N, Walldius G, Bosco C, et al. Serum total bilirubin and risk of cancer: A swedish cohort study and meta‐analysis. Cancers (Basel). 2021;13:5540.

27. Cullinane C, Gillan H, Geraghty J, Evoy D, Rothwell J, Mccartan D, et al. Fertility treatment and breast-cancer incidence: meta-analysis. BJS Open. 2022;6:zrab149.

28. Nouri M, Mohsenpour MA, Katsiki N, Ghobadi S, Jafari A, Faghih S, et al. Effect of Serum Lipid Profile on the Risk of Breast Cancer: Systematic Review and Meta-Analysis of 1,628,871 Women. J Clin Med. 2022;11:4503.

29. Touvier M, Fassier P, His M, Norat T, Chan DSM, Blacher J, et al. Cholesterol and breast cancer risk: A systematic review and meta-analysis of prospective studies. Br J Nutr. 2015;114:347–57.

30. Saadatian-Elahi M, Norat T, Goudable J, Riboli E. Biomarkers of dietary fatty acid intake and the risk of breast cancer: A meta-analysis. Int J Cancer. 2004;111:584–91.

31. Yang B, Ren XL, Wang ZY, Wang L, Zhao F, Guo XJ, et al. Biomarker of long-chain n-3 fatty acid intake and breast cancer: Accumulative evidence from an updated meta-analysis of epidemiological studies. Crit Rev Food Sci Nutr. Taylor & Francis; 2019;59:3152–64.

32. Yang B, Ren XL, Fu YQ, Gao JL, Li D. Ratio of n-3/n-6 PUFAs and risk of breast cancer: A meta-analysis of 274135 adult females from 11 independent prospective studies. BMC Cancer. BMC Cancer; 2014;14:1–14.

33. Anjom-Shoae J, Sadeghi O, Larijani B, Esmaillzadeh A. Dietary intake and serum levels of trans fatty acids and risk of breast cancer: A systematic review and dose-response meta-analysis of prospective studies. Clin Nutr. 2020;39:755–764.

34. Zheng JS, Hu XJ, Zhao YM, Yang J, Li D. Intake of fish and marine n-3 polyunsaturated fatty acids and risk of breast cancer: Meta-analysis of data from 21 independent prospective cohort studies. BMJ. 2013;346:f3706.

35. Cao Y, Hou L, Wang W. Dietary total fat and fatty acids intake, serum fatty acids and risk of breast cancer: A meta-analysis of prospective cohort studies. Int J Cancer. 2016;138:1894–904.

36. Zhou Y, Wang T, Zhai S, Li W, Meng Q. Linoleic acid and breast cancer risk: A meta-analysis. Public Health Nutr. 2016;19:1457–63.

37. Aune D, Chan DSM, Vieira AR, Navarro Rosenblatt DA, Vieira R, Greenwood DC, et al. Dietary compared with blood concentrations of carotenoids and breast cancer risk: a systematic review and meta-analysis of prospective studies. Am J Clin Nutr. Am J Clin Nutr; 2012;96:356–73.

38. Hu F, Wu Z, Li G, Teng C, Liu Y, Wang F, et al. The plasma level of retinol, vitamins A, C and α-tocopherol could reduce breast cancer risk? A meta-analysis and meta-regression. J Cancer Res Clin Oncol. 2015;141:601–14.

39. Wu W, Kang S, Zhang D. Association of vitamin B6, vitamin B12 and methionine with risk of breast cancer: A dose-response meta-analysis. Br J Cancer. Br J Cancer; 2013;109:1926–44.

40. Song D, Deng Y, Liu K, Zhou L, Li N, Zheng Y, et al. Vitamin D intake, blood vitamin D levels, and the risk of breast cancer: a dose-response meta-analysis of observational studies. Aging (Albany NY). 2019;11:12708–32.

41. Hossain S, Beydoun MA, Beydoun HA, Chen X, Zonderman AB, Wood RJ. Vitamin D and breast cancer: A systematic review and meta-analysis of observational studies. Clin Nutr ESPEN. 2019;30:170–84.

42. Zeng J, Gu Y, Fu H, Liu C, Zou Y, Chang H. Association Between One-carbon Metabolism-related Vitamins and Risk of Breast Cancer: A Systematic Review and Meta-analysis of Prospective Studies. Clin Breast Cancer. 2020;20:e469–80.

43. Wulaningsih W, Sagoo HK, Hamza M, Melvin J, Holmberg L, Garmo H, et al. Serum calcium and the risk of breast cancer: Findings from the Swedish AMORIS study and a meta-analysis of prospective studies. Int J Mol Sci. 2016;17:1487.

44. Chang VC, Cotterchio M, Khoo E. Iron intake, body iron status, and risk of breast cancer: a systematic review and meta-analysis. BMC Cancer. BMC Cancer; 2019;19:543.

45. Filippini T, Torres D, Lopes C, Carvalho C, Moreira P, Naska A, et al. Cadmium exposure and risk of breast cancer: A dose-response meta-analysis of cohort studies. Environ Int. 2020;142:105879.

46. Ren X, Xu P, Zhang D, Liu K, Song D, Zheng Y, et al. Association of folate intake and plasma folate level with the risk of breast cancer: a dose-response meta-analysis of observational studies. Aging (Albany NY). 2020;12:21355–75.

47. Tio M, Andrici J, Eslick GD. Folate intake and the risk of breast cancer: A systematic review and meta-analysis. Breast Cancer Res Treat. 2014;145:513–24.

48. Rienks J, Barbaresko J, Nöthlings U. Association of isoflavone biomarkers with risk of chronic disease and mortality: A systematic review and meta-analysis of observational studies. Nutr Rev. 2017;75:616–41.

49. Buck K, Zaineddin AK, Vrieling A, Linseisen J, Chang-Claude J. Meta-analyses of lignans and enterolignans in relation to breast cancer risk. Am J Clin Nutr. 2010;92:141–53.

50. Fu Z, Zhao F, Chen K, Xu J, Li P, Xia D, et al. Association between urinary phthalate metabolites and risk of breast cancer and uterine leiomyoma. Reprod Toxicol. Elsevier Inc.; 2017;74:134–42.

51. Gào X, Wilsgaard T, Jansen EHJM, Holleczek B, Zhang Y, Xuan Y, et al. Pre-diagnostic derivatives of reactive oxygen metabolites and the occurrence of lung, colorectal, breast and prostate cancer: An individual participant data meta-analysis of two large population-based studies. Int J Cancer. 2019;145:49–57.

52. McCormack VA, Dos Santos Silva I. Breast density and parenchymal patterns as markers of breast cancer risk: A meta-analysis. Cancer Epidemiol Biomarkers Prev. 2006;15:1159–69.

53. Antoni S, Sasco AJ, Dos Santos Silva I, McCormack V. Is mammographic density differentially associated with breast cancer according to receptor status? A meta-analysis. Breast Cancer Res Treat. 2013;137:337–47.

54. Bae J, Kim E. Breast Density and Risk of Breast Cancer in Asian Women: A Meta-analysis of Observational Studies. J Prev Med Public Heal. 2016;49:367–75.

55. Warner E, Lockwood G, Tritchler D, Boyd N. The risk of breast cancer associated with mammographic parenchymal patterns: a meta-analysis of the published literature to examine the effect of method of classification. Nutr Rev. 1992;16:67–72.

56. Bond-Smith D, Stone J. Methodological challenges and updated findings from a meta-analysis of the association between mammographic density and breast cancer. Cancer Epidemiol Biomarkers Prev. 2019;28:22–31.

57. Bodewes FTH, van Asselt AA, Dorrius MD, Greuter MJW, de Bock GH. Mammographic breast density and the risk of breast cancer: A systematic review and meta-analysis. Breast. Elsevier Ltd; 2022;66:62–8.

58. Choi YJ, Myung SK, Lee JH. Light alcohol drinking and risk of cancer: A meta-analysis of cohort studies. Cancer Res Treat. 2018;50:474–87.

59. Chen JY, Zhu HC, Guo Q, Shu Z, Bao XH, Sun F, et al. Dose-dependent associations between wine drinking and breast cancer risk - meta-analysis findings. Asian Pacific J Cancer Prev. 2016;17:1221–33.

60. Suzuki R, Orsini N, Mignone L, Saji S, Wolk A. Alcohol intake and risk of breast cancer defined by estrogen and progesterone receptor status - A meta-analysis of epidemiological studies. Int J Cancer. 2008;122:1832–41.

61. Long T, Liu K, Long J, Li J, Cheng L. Dietary glycemic index, glycemic load and cancer risk: a meta-analysis of prospective cohort studies. Eur J Nutr. Springer Berlin Heidelberg; 2022;61:2115–27.

62. Schlesinger S, Chan DSM, Vingeliene S, Vieira AR, Abar L, Polemiti E, et al. Carbohydrates, glycemic index, glycemic load, and breast cancer risk: a systematic review and dose-response meta-analysis of prospective studies. Nutr Rev. 2017;75:420–41.

63. Li N, Guo X, Sun C, Lowe S, Su W, Song Q, et al. Dietary carbohydrate intake is associated with a lower risk of breast cancer: A meta-analysis of cohort studies. Nutr Res. Elsevier Inc.; 2022;100:70–92.

64. Llaha F, Gil-Lespinard M, Unal P, de Villasante I, Castañeda J, Zamora-Ros R. Consumption of sweet beverages and cancer risk. A systematic review and meta-analysis of observational studies. Nutrients. 2021;13:1–35.

65. Gao Y, Huang YB, Liu XO, Chen C, Dai HJ, Song FJ, et al. Tea consumption, alcohol drinking and physical activity associations with breast cancer risk among Chinese females: A systematic review and meta-analysis. Asian Pacific J Cancer Prev. 2013;14:7543–50.

66. Bamia C, Turati F, Guha N, van den Brandt P, Loomis D, Ferraroni M, et al. The role of coffee consumption in breast and ovarian cancer risk: Updated meta-analyses. Epidemiol Biostat Public Heal. 2019;16:e13078.

67. Wang Y, Zhao Y, Chong F, Song M, Sun Q, Li T, et al. A dose-response meta-analysis of green tea consumption and breast cancer risk. Int J Food Sci Nutr. Taylor & Francis; 2020;71:656–67.

68. Dong JY, Zhang L, He K, Qin LQ. Dairy consumption and risk of breast cancer: A meta-analysis of prospective cohort studies. Breast Cancer Res Treat. 2011;127:23–31.

69. Kazemi A, Barati-Boldaji R, Soltani S, Mohammadipoor N, Esmaeilinezhad Z, Clark CCT, et al. Intake of various food groups and risk of breast cancer: A systematic review and dose-response meta-analysis of prospective studies. Adv Nutr. Oxford University Press; 2021;12:809–49.

70. Wu J, Zeng R, Huang J, Li X, Zhang J, Ho JCM, et al. Dietary protein sources and incidence of breast cancer: A dose-response meta-analysis of prospective studies. Nutrients. 2016;8:730.

71. Zang J, Shen M, Du S, Chen T, Zou S. The association between dairy intake and breast cancer in western and Asian populations: A systematic review and meta-analysis. J Breast Cancer. 2015;18:313–22.

72. Xiao Y, Xia J, Li L, Ke Y, Cheng J, Xie Y, et al. Associations between dietary patterns and the risk of breast cancer: A systematic review and meta-analysis of observational studies. Breast Cancer Res. Breast Cancer Research; 2019;21:16.

73. Schwingshackl L, Schwedhelm C, Galbete C, Hoffmann G. Adherence to mediterranean diet and risk of cancer: An updated systematic review and meta-analysis. Nutrients. 2017;9:1561–86.

74. Brennan S, Cantwell MM, Cardwell CR, Velentzis LS, Woodside J V. Dietary patterns and breast cancer risk: A systematic review. Am J Clin Nutr. 2010;91:1294–302.

75. Hayati Z, Jafarabadi MA, Pirouzpanah S. Dietary inflammatory index and breast cancer risk: an updated meta-analysis of observational studies. Eur J Clin Nutr. Springer US; 2022;76:1073–87.

76. Elands RJJ, Simons CCJM, Van Dongen M, Schouten LJ, Verhage BJ, Van Den Brandt PA, et al. A systematic literature review and meta-regression analysis on early-life energy restriction and cancer risk in humans. PLoS One. 2016;11:e0158003.

77. Parra-Soto S, Ahumada D, Petermann-Rocha F, Boonpoor J, Gallegos JL, Anderson J, et al. Association of meat, vegetarian, pescatarian and fish-poultry diets with risk of 19 cancer sites and all cancer: findings from the UK Biobank prospective cohort study and meta-analysis. BMC Med. BioMed Central; 2022;20:79.

78. Godos J, Bella F, Sciacca S, Galvano F, Grosso G. Vegetarianism and breast, colorectal and prostate cancer risk: an overview and meta-analysis of cohort studies. J Hum Nutr Diet. J Hum Nutr Diet; 2017;30:349–59.

79. Dinu M, Abbate R, Gensini GF, Casini A, Sofi F. Vegetarian, vegan diets and multiple health outcomes: A systematic review with meta-analysis of observational studies. Crit Rev Food Sci Nutr. Crit Rev Food Sci Nutr; 2017;57:3640–9.

80. Li C, Yang L, Zhang D, Jiang W. Systematic review and meta-analysis suggest that dietary cholesterol intake increases risk of breast cancer. Nutr Res. Elsevier Inc.; 2016;36:627–35.

81. Liu J, Li X, Hou J, Sun J, Guo N, Wang Z. Dietary Intake of N-3 and N-6 Polyunsaturated Fatty Acids and Risk of Cancer: Meta-Analysis of Data from 32 Studies. Nutr Cancer. Taylor & Francis; 2021;73:901–13.

82. Nindrea RD, Aryandono T, Lazuardi L, Dwiprahasto I. Association of dietary intake ratio of n-3/n-6 polyunsaturated fatty acids with breast cancer risk in Western and Asian countries: A meta-analysis. Asian Pacific J Cancer Prev. 2019;20:1321–7.

83. Xia H, Ma S, Wang S, Sun G. Meta-analysis of saturated fatty acid intake and breast cancer risk. Med (United States). 2015;94:e2391.

84. Michels N, Specht IO, Heitmann BL, Chajès V, Huybrechts I. Dietary trans-fatty acid intake in relation to cancer risk: A systematic review and meta-analysis. Nutr Rev. 2021;79:758–76.

85. Farvid MS, Sidahmed E, Spence ND, Mante Angua K, Rosner BA, Barnett JB. Consumption of red meat and processed meat and cancer incidence: a systematic review and meta-analysis of prospective studies. Eur J Epidemiol. Springer Netherlands; 2021;36:937–51.

86. Rezaianzadeh A, Ghorbani M, Rezaeian S, Kassani A. Red meat consumption and breast cancer risk in premenopausal women: A systematic review and meta-analysis. Middle East J Cancer. 2018;9:5–12.

87. Zhihui W, Weihua Y, Zupei W, Jinlin H. Fish consumption and risk of breast cancer: meta-analysis of 27 observational studies. Nutr Hosp. 2016;33:703–12.

88. Keum N, Lee DH, Marchand N, Oh H, Liu H, Aune D, et al. Egg intake and cancers of the breast, ovary and prostate: A dose-response meta-analysis of prospective observational studies. Br J Nutr. 2015;114:1099–107.

89. Si R, Qu K, Jiang Z, Yang X, Gao P. Egg consumption and breast cancer risk: A meta-analysis. Breast Cancer. 2014;21:251–61.

90. Farvid MS, Stern MC, Norat T, Sasazuki S, Vineis P, Weijenberg MP, et al. Consumption of red and processed meat and breast cancer incidence: A systematic review and meta-analysis of prospective studies. Int J Cancer. 2018;143:2787–99.

91. Hidayat K, Chen GC, Zhang R, Du X, Zou SY, Shi BM, et al. Calcium intake and breast cancer risk: Meta-analysis of prospective cohort studies. Br J Nutr. 2016;116:158–66.

92. Cai X, Wang C, Yu W, Fan W, Wang S, Shen N, et al. Selenium Exposure and Cancer Risk: an Updated Meta-analysis and Meta-regression. Sci Rep. Sci Rep; 2016;6:19213.

93. Adani G, Filippini T, Wise LA, Halldorsson TI, Blaha L, Vinceti M. Dietary intake of acrylamide and risk of breast, endometrial, and ovarian cancers: a systematic review and dose-response meta-analysis. Cancer Epidemiol Biomarkers Prev. 2020;29:1095–106.

94. Fulan H, Changxing J, Yi Baina W, Wencui Z, Chunqing L, Fan W, et al. Retinol, vitamins A, C, and E and breast cancer risk: A meta-analysis and meta-regression. Cancer Causes Control. Springer; 2011;22:1383–96.

95. Hu F, Wang Yi B, Zhang W, Liang J, Lin C, Li D, et al. Carotenoids and breast cancer risk: a meta-analysis and meta-regression. Breast Cancer Res Treat. Breast Cancer Res Treat; 2012;131:239–53.

96. Yu L, Tan Y, Zhu L. Dietary vitamin B2 intake and breast cancer risk: a systematic review and meta-analysis. Arch Gynecol Obstet. Arch Gynecol Obstet; 2017;295:721–9.

97. Zhang D, Xu P, Li Y, Wei B, Yang S, Zheng Y, et al. Association of vitamin C intake with breast cancer risk and mortality: A meta-analysis of observational studies. Aging (Albany NY). 2020;12:18415–35.

98. Estébanez N, Gómez-Acebo I, Palazuelos C, Llorca J, Dierssen-Sotos T. Vitamin D exposure and Risk of Breast Cancer: A meta-analysis. Sci Rep. Sci Rep; 2018;8:9039.

99. Chan ALF, Leung HWC, Wang SF. Multivitamin supplement use and risk of breast cancer: A meta-analysis. Ann Pharmacother. SAGE PublicationsSage CA: Los Angeles, CA; 2011;45:476–84.

100. Aune D, Chan DSM, Greenwood DC, Vieira AR, Navarro Rosenblatt DA, Vieira R, et al. Dietary fiber and breast cancer risk: A systematic review and meta-analysis of prospective studies. Ann Oncol. 2012;23:1394–402.

101. Farvid MS, Spence ND, Holmes MD, Barnett JB. Fiber consumption and breast cancer incidence: A systematic review and meta-analysis of prospective studies. Cancer. 2020;126:3061–75.

102. Grosso G, Godos J, Lamuela-Raventos R, Ray S, Micek A, Pajak A, et al. A comprehensive meta-analysis on dietary flavonoid and lignan intake and cancer risk: Level of evidence and limitations. Mol Nutr Food Res. Wiley-VCH Verlag; 2017;61:2–12.

103. Liu F, Peng Y, Qiao Y, Huang Y, Song F, Zhang M, et al. Consumption of flavonoids and risk of hormone-related cancers: a systematic review and meta-analysis of observational studies. Nutr J. BioMed Central; 2022;21:1–13.

104. Zhao TT, Jin F, Li JG, Xu YY, Dong HT, Liu Q, et al. Dietary isoflavones or isoflavone-rich food intake and breast cancer risk: A meta-analysis of prospective cohort studies. Clin Nutr. Elsevier Ltd; 2019;38:136–45.

105. Aune D, Chan DSM, Vieira AR, Navarro Rosenblatt DA, Vieira R, Greenwood DC, et al. Fruits, vegetables and breast cancer risk: a systematic review and meta-analysis of prospective studies. Breast Cancer Res Treat. Breast Cancer Res Treat; 2012;134:479–93.

106. Song JK, Bae JM. Citrus fruit intake and breast cancer risk: a quantitative systematic review. J Breast Cancer. J Breast Cancer; 2013;16:72–6.

107. Chen H, Shao F, Zhang F, Miao Q. Association between dietary carrot intake and breast cancer: A meta-analysis. Med. 2018;97:e12164.

108. Liu X, Lv K. Cruciferous vegetables intake is inversely associated with risk of breast cancer: A meta-analysis. Breast. 2013;22:309–13.

109. Zhang J, Yang J. Allium Vegetables Intake and Risk of Breast Cancer: A Meta-Analysis. Iran J Public Health. 2022;51:746–57.

110. Li J, Zou L, Chen W, Zhu B, Shen N, Ke J, et al. Dietary mushroom intake may reduce the risk of breast cancer: evidence from a meta-analysis of observational studies. PLoS One. PLoS One; 2014;9:e93437.

111. Zhang D, Dai C, Zhou L, Li Y, Liu K, Deng YJ, et al. Meta-analysis of the association between nut consumption and the risks of cancer incidence and cancer-specific mortality. Aging (Albany NY). 2020;12:10772–94.

112. Wei Y, Lv J, Guo Y, Bian Z, Gao M, Du H, et al. Soy intake and breast cancer risk: a prospective study of 300,000 Chinese women and a dose–response meta-analysis. Eur J Epidemiol. 2020;35:567–78.

113. Wang Q, Liu X, Ren S. Tofu intake is inversely associated with risk of breast cancer: A meta-analysis of observational studies. PLoS One. 2020;15:1–13.

114. Qin LQ, Xu JY, Wang PY, Hoshi K. Soyfood intake in the prevention of breast cancer risk in women: A meta-analysis of observational epidemiological studies. J Nutr Sci Vitaminol (Tokyo). 2006;52:428–36.

115. Markellos C, Ourailidou ME, Gavriatopoulou M, Halvatsiotis P, Sergentanis TN, Psaltopoulou T. Olive oil intake and cancer risk: A systematic review and meta-analysis. PLoS One. 2022;17:e0261649.

116. Xiao Y, Ke Y, Wu S, Huang S, Li S, Lv Z, et al. Association between whole grain intake and breast cancer risk: a systematic review and meta-analysis of observational studies. Nutr J. Nutr J; 2018;17:87.

117. Woo HD, Park S, Oh K, Kim HJ, Shin HR, Moon HK, et al. Diet and cancer risk in the Korean population: A meta-analysis. Asian Pacific J Cancer Prev. Asian Pacific Journal of Cancer Prevention; 2014;15:8509–19.

118. Wei W, Wu BJ, Wu Y, Tong ZT, Zhong F, Hu CY. Association between long-term ambient air pollution exposure and the risk of breast cancer: a systematic review and meta-analysis. Environ Sci Pollut Res. Environmental Science and Pollution Research; 2021;28:63278–96.

119. Zhang Z, Yan W, Chen Q, Zhou N, Xu Y. The relationship between exposure to particulate matter and breast cancer incidence and mortality: A meta-analysis. Med (United States). 2019;98:e18349.

120. Liu G, Cai W, Liu H, Jiang H, Bi Y, Wang H. The Association of Bisphenol A and Phthalates with Risk of Breast Cancer : A Meta-Analysis. Int J Environ Res Public Health. 2021;18:2375.

121. Zhang Y, Lai J, Ruan G, Chen C, Wang DW. Meta-analysis of extremely low frequency electromagnetic fields and cancer risk: a pooled analysis of epidemiologic studies. Environ Int. Environ Int; 2016;88:36–43.

122. Erren T. A meta-analysis of epidemiologic studies of electric and magnetic fields and breast cancer in women and men. Bioelectromagnetics. 2001;Suppl 5:S105-19.

123. Hiller TWR, O’sullivan DE, Brenner DR, Peters CE, King WD. Solar ultraviolet radiation and breast cancer risk: A systematic review and meta-analysis. Environ Health Perspect. 2020;128:1–11.

124. Shamshirian A, Heydari K, Shams Z, Aref AR, Shamshirian D, Tamtaji OR, et al. Breast cancer risk factors in Iran: A systematic review & Meta-analysis. Horm Mol Biol Clin Investig. 2020;41.

125. Akinyemiju TF, Genkinger JM, Farhat M, Wilson A, Gary-Webb TL, Tehranifar P. Residential environment and breast cancer incidence and mortality: A systematic review and meta-analysis. BMC Cancer. 2015;15:191.

126. Lee PN, Hamling JS. Environmental tobacco smoke exposure and risk of breast cancer in nonsmoking women. An updated review and meta-analysis. Inhal Toxicol. 2016;28:431–54.

127. Leng L, Li J, Luo X mei, Kim J young, Li Y meng, Guo X mei, et al. Polychlorinated biphenyls and breast cancer: A congener-specific meta-analysis. Environ Int. Environ Int; 2016;88:133–41.

128. Zhang J, Huang Y, Wang X, Lin K, Wu K. Environmental polychlorinated biphenyl exposure and breast cancer risk: A meta-analysis of observational studies. PLoS One. PLoS One; 2015;10.

129. Xu S, Wang H, Liu Y, Zhang C, Xu Y, Tian F, et al. Hair chemicals may increase breast cancer risk: A meta-Analysis of 210319 subjects from 14 studies. PLoS One. 2021;16:1–17.

130. Ingber SZ, Buser MC, Pohl HR, Abadin HG, Edward Murray H, Scinicariello F. DDT/DDE and breast cancer: A meta-analysis. Regul Toxicol Pharmacol. Elsevier Inc.; 2013;67:421–33.

131. Liu T, Xu QE, Zhang CH, Zhang P. Occupational exposure to methylene chloride and risk of cancer: a meta-analysis. Cancer Causes Control. Cancer Causes Control; 2013;24:2037–49.

132. Jiang H, Liu H, Liu G, Yu J, Liu N, Jin Y, et al. Associations between Polyfluoroalkyl Substances Exposure and Breast Cancer: A Meta-analysis. Toxics. 2022;10:318.

133. Anothaisintawee T, Wiratkapun C, Lerdsitthichai P, Kasamesup V, Wongwaisayawan S, Srinakarin J, et al. Risk factors of breast cancer: A systematic review and meta-analysis. Asia-Pacific J Public Heal. 2013;25:368–87.

134. Kim S, Ko Y, Lee HJ, Lim J eun. Menopausal hormone therapy and the risk of breast cancer by histological type and race: a meta-analysis of randomized controlled trials and cohort studies. Breast Cancer Res Treat. Springer US; 2018;170:667–75.

135. Ni XJ, Xia TS, Zhao YC, Ma JJ, Zhao J, Liu XA, et al. Postmenopausal hormone therapy is associated with in situ breast cancer risk. Asian Pacific J Cancer Prev. 2012;13:3917–25.

136. Sergentanis TN, Diamantaras AA, Perlepe C, Kanavidis P, Skalkidou A, Petridou ET. IVF and breast cancer: a systematic review and meta-analysis. Hum Reprod Update. Hum Reprod Update; 2014;20:106–23.

137. Kahlenborn C, Modugno F, Potter DM, Severs WB. Oral contraceptive use as a risk factor for premenopausal breast cancer: A meta-analysis. Mayo Clin Proc. 2006;81:1290–302.

138. Conz L, Mota BS, Bahamondes L, Teixeira Dória M, Françoise Mauricette Derchain S, Rieira R, et al. Levonorgestrel-releasing intrauterine system and breast cancer risk: A systematic review and meta-analysis. Acta Obstet Gynecol Scand. 2020;99:970–82.

139. Ji LW, Jing CX, Zhuang SL, Pan WC, Hu XP. Effect of age at first use of oral contraceptives on breast cancer risk: An updated meta-analysis. Med. 2019;98:e15719.

140. Moorman PG, Havrilesky LJ, Gierisch JM, Coeytaux RR, Lowery WJ, Urrutia RP, et al. Oral contraceptives and risk of ovarian cancer and breast cancer among high-risk women: A systematic review and meta-analysis. J Clin Oncol. 2013;31:4188–98.

141. Hardefeldt PJ, Penninkilampi R, Edirimanne S, Eslick GD. Physical Activity and Weight Loss Reduce the Risk of Breast Cancer: A Meta-analysis of 139 Prospective and Retrospective Studies. Clin Breast Cancer. Elsevier Inc.; 2018;18:e601–12.

142. Hidayat K, Zhou HJ, Shi BM. Influence of physical activity at a young age and lifetime physical activity on the risks of 3 obesity-related cancers: Systematic review and meta-analysis of observational studies. Nutr Rev. 2020;78:1–18.

143. Raza W, Krachler B, Forsberg B, Sommar JN. Health benefits of leisure time and commuting physical activity: A meta-analysis of effects on morbidity. J Transp Heal. Elsevier Ltd; 2020;18:100873.

144. Neilson HK, Farris MS, Stone CR, Vaska MM, Brenner DR, Friedenreich CM. Moderate-vigorous recreational physical activity and breast cancer risk, stratified by menopause status: A systematic review and meta-analysis. Menopause. 2016;24:322–44.

145. Zhou Y, Zhao H, Peng C. Association of sedentary behavior with the risk of breast cancer in women: Update meta-analysis of observational studies. Ann Epidemiol. Elsevier Inc; 2015;25:687–97.

146. Chong F, Wang Y, Song M, Sun Q, Xie W, Song C. Sedentary behavior and risk of breast cancer: a dose–response meta-analysis from prospective studies. Breast Cancer. Springer Japan; 2021;28:48–59.

147. He C, Anand ST, Ebell MH, Vena JE, Robb SW. Circadian disrupting exposures and breast cancer risk: a meta-analysis. Int Arch Occup Environ Health. Springer Berlin Heidelberg; 2015;88:533–47.

148. Urbano T, Vinceti M, Wise LA, Filippini T. Light at night and risk of breast cancer: a systematic review and dose–response meta-analysis. Int J Health Geogr. BioMed Central; 2021;20:1–26.

149. Van NTH, Hoang T, Myung SK. Night shift work and breast cancer risk: A meta-analysis of observational epidemiological studies. Carcinogenesis. 2021;42:1260–9.

150. Schwarz C, Pedraza-Flechas AM, Pastor-Barriuso R, Lope V, de Larrea NF, Jiménez-Moleón JJ, et al. Long-term nightshift work and breast cancer risk: An updated systematic review and meta-analysis with special attention to menopausal status and to recent nightshift work. Cancers (Basel). 2021;13:5952.

151. Hong J, He Y, Fu R, Si Y, Xu B, Xu J, et al. The relationship between night shift work and breast cancer incidence: A systematic review and meta-analysis of observational studies. Open Med. 2022;12:712–31.

152. Ijaz S, Verbeek J, Seidler A, Lindbohm ML, Ojajärvi A, Orsini N, et al. Night-shift work and breast cancer - A systematic review and meta-analysis. Scand J Work Environ Heal. 2013;39:431–47.

153. Qin Y, Zhou Y, Zhang X, Wei X, He J. Sleep duration and breast cancer risk: A meta-analysis of observational studies. Int J Cancer. 2014;134:1166–73.

154. Wong ATY, Heath AK, Tong TYN, Reeves GK, Floud S, Beral V, et al. Sleep duration and breast cancer incidence: results from the Million Women Study and meta-analysis of published prospective studies. Sleep. 2021;44:zsaa166.

155. Macacu A, Autier P, Boniol M, Boyle P. Active and passive smoking and risk of breast cancer: a meta-analysis. Breast Cancer Res Treat. Springer US; 2015;154:213–24.

156. Lundqvist A, Andersson E, Ahlberg I, Nilbert M, Gerdtham U. Socioeconomic inequalities in breast cancer incidence and mortality in Europe - A systematic review and meta-analysis. Eur J Public Health. 2016;26:804–13.

157. Dong JY, Qin LQ. Education level and breast cancer incidence: A meta-analysis of cohort studies. Menopause. 2020;27:113–8.

158. Li M, Han M, Chen Z, Tang Y, Ma J, Zhang Z, et al. Does marital status correlate with the female breast cancer risk? A systematic review and meta-analysis of observational studies. PLoS One. 2020;15:1–17.

159. Santos MCL, Horta BL, Amaral JJF do, Fernandes PFCBC, Galvão CM, Fernandes AFC. Association between stress and breast cancer in women: a meta-analysis. Cad Saude Publica. 2009;25:S453–63.

160. Bahri N, Fathi Najafi T, Homaei Shandiz F, Tohidinik HR, Khajavi A. The relation between stressful life events and breast cancer: a systematic review and meta-analysis of cohort studies. Breast Cancer Res Treat. Springer US; 2019;176:53–61.

161. Turati F, Dalmartello M, Bravi F, Serraino D, Augustin L, Giacosa A, et al. Adherence to the World Cancer Research Fund/American Institute for Cancer Research Recommendations and the Risk of Breast Cancer. Nutrients. Multidisciplinary Digital Publishing Institute; 2020;12:607.

162. Hardefeldt PJ, Edirimanne S, Eslick GD. Diabetes increases the risk of breast cancer: a meta-analysis. Endocr Relat Cancer. 2012;19:793–803.

163. Seretis A, Cividini S, Markozannes G, Tseretopoulou X, Lopez DS, Ntzani EE, et al. Association between blood pressure and risk of cancer development: a systematic review and meta-analysis of observational studies. Sci Rep. Springer US; 2019;9:1974.

164. Guo M, Liu T, Li P, Wang T, Zeng C, Yang M, et al. Association Between Metabolic Syndrome and Breast Cancer Risk: An Updated Meta-Analysis of Follow-Up Studies. Front Oncol. 2019;9:1290.

165. Tran TVT, Kitahara CM, Leenhardt L, de Vathaire F, Boutron-Ruault MC, Journy N. The effect of thyroid dysfunction on breast cancer risk: an updated meta-analysis. Endocr Relat Cancer. 2023;30:1–42.

166. Bonifazi M, Tramacere I, Pomponio G, Gabrielli B, Avvedimento E V., La Vecchia C, et al. Systemic sclerosis (scleroderma) and cancer risk: Systematic review and meta-analysis of observational studies. Rheumatol (United Kingdom). 2013;52:143–54.

167. Song L, Wang Y, Zhang J, Song N, Xu X, Lu Y. The risks of cancer development in systemic lupus erythematosus (SLE) patients: A systematic review and meta-analysis. Arthritis Res Ther. BioMed Central Ltd.; 2018;20:270.

168. Catalá-López F, Suárez-Pinilla M, Suárez-Pinilla P, Valderas JM, Gómez-Beneyto M, Martinez S, et al. Inverse and direct cancer comorbidity in people with central nervous system disorders: A meta-analysis of cancer incidence in 577,013 participants of 50 observational studies. Psychother Psychosom. 2014;83:89–105.

169. Hesari E, Ahmadinezhad M, Arshadi M, Azizi H, Khodamoradi F. The association between migraine and breast cancer risk: A systematic review and meta-analysis. PLoS One. 2022;17:1–13.

170. Zhang P, Liu B. Association between Parkinson’s Disease and Risk of Cancer: A PRISMA-compliant Meta-analysis. ACS Chem Neurosci. 2019;10:4430–9.

171. Sun HL, Dong XX, Cong YJ, Gan Y, Deng J, Cao SY, et al. Depression and the risk of breast cancer: A meta-analysis of cohort studies. Asian Pacific J Cancer Prev. 2015;16:3233–9.

172. Chen JH, Yuan Q, Ma YN, Zhen SH, Wen DL. Relationship between bone mineral density and the risk of breast cancer: A systematic review and dose–response meta-analysis of ten cohort studies. Cancer Manag Res. 2019;11:1453–64.

173. Lovrics O, Butt J, Lee Y, Lovrics P, Boudreau V, Anvari M, et al. The effect of bariatric surgery on breast cancer incidence and characteristics: A meta-analysis and systematic review. Am J Surg. Elsevier Ltd; 2021;222:715–22.

174. Wang Y, Song Z, Zhang S, Wang X, Li P. Risk-reducing salpingo-oophorectomy and breast cancer risk in BRCA1 or BRCA2 mutation carriers: A systematic review and meta-analysis. Eur J Surg Oncol. Elsevier Ltd, BASO ~ The Association for Cancer Surgery, and the European Society of Surgical Oncology; 2022;48:1209–16.

175. Shao J, Wu L, Leng WD, Fang C, Zhu YJ, Jin YH, et al. Periodontal disease and breast cancer: A meta-analysis of 1,73,162 participants. Front Oncol. 2018;8:601.

176. Zhou Y, Li W, Herath C, Xia J, Hu B, Song F, et al. Off-hour admission and mortality risk for 28 specific diseases: A systematic review and meta-analysis of 251 cohorts. J Am Heart Assoc. John Wiley and Sons Inc.; 2016;5:e003102.

177. Vojtechova P, Martin RM. The association of atopic diseases with breast, prostate, and colorectal cancers: A meta-analysis. Cancer Causes Control. 2009;20:1091–105.

178. Overbeek JA, Bakker M, van der Heijden AAWA, van Herk-Sukel MPP, Herings RMC, Nijpels G. Risk of dipeptidyl peptidase-4 (DPP-4) inhibitors on site-specific cancer: A systematic review and meta-analysis. Diabetes Metab Res Rev. Diabetes Metab Res Rev; 2018;34:e3004.

179. Colmers IN, Bowker SL, Tjosvold LA, Johnson JA. Insulin use and cancer risk in patients with type 2 diabetes: a systematic review and meta-analysis of observational studies. Diabetes Metab. Diabetes Metab; 2012;38:485–506.

180. Tang GH, Satkunam M, Pond GR, Steinberg GR, Blandino G, Schunemann HJ, et al. Association of Metformin with Breast Cancer Incidence and Mortality in Patients with Type II Diabetes: A GRADE-Assessed Systematic Review and Meta-analysis. Cancer Epidemiol Biomarkers Prev. Cancer Epidemiol Biomarkers Prev; 2018;27:627–35.

181. Du R, Lin L, Cheng D, Xu Y, Xu M, Chen Y, et al. Thiazolidinedione therapy and breast cancer risk in diabetic women: A systematic review and meta-analysis. Diabetes Metab Res Rev. 2018;34:9–11.

182. Xie Y, Wang M, Xu P, Deng Y, Zheng Y, Yang S, et al. Association Between Antihypertensive Medication Use and Breast Cancer: A Systematic Review and Meta-Analysis. Front Pharmacol. 2021;12:609901.

183. Zhang C, Xie SH, Xu B, Lu S, Liu P. Digitalis Use and the Risk of Breast Cancer: A Systematic Review and Meta-Analysis. Drug Saf. Springer International Publishing; 2017;40:285–92.

184. Osman MH, Farrag E, Selim M, Osman MS, Hasanine A, Selim A. Cardiac glycosides use and the risk and mortality of cancer; Systematic review and meta-Analysis of observational studies. PLoS One. 2017;12:e0178611.

185. Ni H, Rui Q, Zhu X, Yu Z, Gao R, Liu H. Antihypertensive drug use and breast cancer risk: A metaanalysis of observational studies. Oncotarget. Oncotarget; 2017;8:62545–60.

186. Bommareddy K, Hamade H, Lopez-Olivo MA, Wehner M, Tosh T, Barbieri JS. Association of Spironolactone Use with Risk of Cancer: A Systematic Review and Meta-analysis. JAMA Dermatology. 2022;158:275–82.

187. Zhao G, Ji Y, Ye Q, Ye X, Wo G, Chen X, et al. Effect of statins use on risk and prognosis of breast cancer: a meta-analysis. Anticancer Drugs. 2022;33:E507–18.

188. Ma S, Guo C, Sun C, Han T, Zhang H, Qu G, et al. Aspirin Use and Risk of Breast Cancer: A Meta-analysis of Observational Studies from 1989 to 2019. Clin Breast Cancer. Elsevier Inc.; 2021;21:552–65.

189. Takkouche B, Regueira-Méndez C, Etminan M. Breast cancer and use of nonsteroidal anti-inflammatory drugs: A meta-analysis. J Natl Cancer Inst. 2008;100:1439–47.

190. Lu L, Shi L, Zeng J, Wen Z. Aspirin as a potential modality for the chemoprevention of breast cancer: A dose-response meta-analysis of cohort studies from 857,831 participants. Oncotarget. Oncotarget; 2017;8:40389–401.

191. Zhong S, Chen L, Zhang X, Yu D, Tang J, Zhao J. Aspirin use and risk of breast cancer: Systematic review and meta-analysis of observational studies. Cancer Epidemiol Biomarkers Prev. 2015;24:1645–55.

192. Peng R, Liang X, Zhang G, Yao Y, Chen Z, Pan X, et al. Association Use of Bisphosphonates with Risk of Breast Cancer: A Meta-Analysis. Biomed Res Int. 2020;2020:5606573.

193. Liu Y, Zhang X, Sun H, Zhao S, Zhang Y, Li D, et al. Bisphosphonates and primary breast cancer risk: An updated systematic review and meta-analysis involving 963,995 women. Clin Epidemiol. 2019;11:593–603.

194. Simin J, Tamimi RM, Engstrand L, Callens S, Brusselaers N. Antibiotic use and the risk of breast cancer: A systematic review and dose-response meta-analysis. Pharmacol Res. Elsevier; 2020;160:105072.

195. Leung JCN, Ng DWY, Chu RYK, Chan EWW, Huang L, Lum DH, et al. Association of antipsychotic use with breast cancer: a systematic review and meta-analysis of observational studies with over 2 million individuals-CORRIGENDUM. Epidemiol Psychiatr Sci. 2022;31:e66.

196. Song HJ, Jeon N, Squires P. The association between acid-suppressive agent use and the risk of cancer: a systematic review and meta-analysis. Eur J Clin Pharmacol. European Journal of Clinical Pharmacology; 2020;76:1437–56.

197. Chen LX, Ning GZ, Zhou ZR, Li YL, Zhang D, Wu QL, et al. The carcinogenicity of alendronate in patients with osteoporosis: evidence from cohort studies. PLoS One. PLoS One; 2015;10:e0123080.

198. Li R, Li X, Yan P, Bing Z, Cao L, Hui X, et al. Relationship between antidepressive agents and incidence risk of breast cancer: Systematic review and meta-analysis. Futur Oncol. 2021;17:1105–24.

199. Angelousi AG, Anagnostou VK, Stamatakos MK, Georgiopoulos GA, Kontzoglou KC. Primary HT and risk for breast cancer: A systematic review and meta-analysis. Eur. J. Endocrinol. Eur J Endocrinol; 2012. p. 373–81.

200. Lambertini M, Santoro L, Del Mastro L, Nguyen B, Livraghi L, Ugolini D, et al. Reproductive behaviors and risk of developing breast cancer according to tumor subtype: A systematic review and meta-analysis of epidemiological studies. Cancer Treat Rev. Elsevier Ltd; 2016;49:65–76.

201. Tong H, Wu Y, Yan Y, Dong Y, Guan X, Liu Y, et al. No association between abortion and risk of breast cancer among nulliparous women: Evidence from a meta-analysis. Med (United States). 2020;99:E20251.

202. Guo J, Huang Y, Yang L, Xie Z, Song S, Yin J, et al. Association between abortion and breast cancer: an updated systematic review and meta-analysis based on prospective studies. Cancer Causes Control. Springer International Publishing; 2015;26:811–9.

203. Ma H, Bernstein L, Pike MC, Ursin G. Reproductive factors and breast cancer risk according to joint estrogen and progesterone receptor status: A meta-analysis of epidemiological studies. Breast Cancer Res. 2006;8:R43.

204. Nagata C, Hu YH, Shimizu H. Effects of menstrual and reproductive factors on the risk of breast cancer: Meta-analysis of the case-control studies in Japan. Japanese J Cancer Res. 1995;86:910–5.

205. Gaudet MM, Patel A V., Sun J, Teras LR, Gapstur SM. Tubal sterilization and breast cancer incidence: Results from the cancer prevention study II nutrition cohort and meta-analysis. Am J Epidemiol. Am J Epidemiol; 2013;177:492–9.

206. Li C, Fan Z, Lin X, Cao M, Song F, Song F. Parity and risk of developing breast cancer according to tumor subtype: A systematic review and meta-analysis. Cancer Epidemiol. Elsevier Ltd; 2021;75:102050.

207. Unar-Munguía M, Torres-Mejía G, Colchero MA, González De Cosío T. Breastfeeding Mode and Risk of Breast Cancer: A Dose-Response Meta-Analysis. J Hum Lact. 2017;33:422–34.

208. Bernier MO, Bossard N, Ayzac L, Thalabard JC. Breastfeeding and risk of breast cancer: A meta-analysis of published studies. Hum Reprod Update. 2000;6:374–86.

209. Islami F, Liu Y, Jemal A, Zhou J, Weiderpass E, Colditz G, et al. Breastfeeding and breast cancer risk by receptor status-a systematic review and meta-analysis. Ann Oncol. 2015;26:2398–407.

210. Wang F, Zhang W, Cheng W, Huo N, Zhang S. Preeclampsia and cancer risk in women in later life: a systematic review and meta-analysis of cohort studies. Menopause. 2021;28:1070–8.

211. Sun M, Fan Y, Hou Y, Fan Y. Preeclampsia and maternal risk of breast cancer: a meta-analysis of cohort studies. J Matern Fetal Neonatal Med. J Matern Fetal Neonatal Med; 2018;31:2484–91.

212. Xie C, Wang W, Li X, Shao N, Li W. Gestational diabetes mellitus and maternal breast cancer risk: a meta-analysis of the literature. J Matern Neonatal Med. 2019;32:1022–32.
